# The role of ZAP and OAS3/RNAseL pathways in the attenuation of an RNA virus with elevated frequencies of CpG and UpA dinucleotides

**DOI:** 10.1093/nar/gkz581

**Published:** 2019-07-05

**Authors:** Valerie Odon, Jelke J Fros, Niluka Goonawardane, Isabelle Dietrich, Ahmad Ibrahim, Kinda Alshaikhahmed, Dung Nguyen, Peter Simmonds

**Affiliations:** 1 Nuffield Department of Medicine, Peter Medawar Building for Pathogen Research, University of Oxford, Oxford OX1 3SY, UK; 2 Laboratory of Virology, Wageningen University, Droevendaalsesteeg 1, 6708 PB Wageningen, The Netherlands

## Abstract

Zinc finger antiviral protein (ZAP) is a powerful restriction factor for viruses with elevated CpG dinucleotide frequencies. We report that ZAP similarly mediates antiviral restriction against echovirus 7 (E7) mutants with elevated frequencies of UpA dinucleotides. Attenuation of both CpG- and UpA-high viruses and replicon mutants was reversed in ZAP k/o cell lines, and restored by plasmid-derived reconstitution of expression in k/o cells. In pull-down assays, ZAP bound to viral RNA transcripts with either CpG- and UpA-high sequences inserted in the R2 region. We found no evidence that attenuation of CpG- or UpA-high mutants was mediated through either translation inhibition or accelerated RNA degradation. Reversal of the attenuation of CpG-high, and UpA-high E7 viruses and replicons was also achieved through knockout of RNAseL and oligodenylate synthetase 3 (OAS3), but not OAS1. WT levels of replication of CpG- and UpA-high mutants were observed in OAS3 k/o cells despite abundant expression of ZAP, indicative of synergy or complementation of these hitherto unconnected pathways. The dependence on expression of ZAP, OAS3 and RNAseL for CpG/UpA-mediated attenuation and the variable and often low level expression of these pathway proteins in certain cell types, such as those of the central nervous system, has implications for the use of CpG-elevated mutants as attenuated live vaccines against neurotropic viruses.

## INTRODUCTION

RNA viruses infecting vertebrates possess relatively small, highly compact genomes that encode an extremely limited number of structural and replication-associated proteins. Despite their apparent simplicity, their evolutionary success depends critically on their abilities not only to replicate their genomes and transmit between cells and hosts, but also to circumvent or counteract a battery of cellular and whole host defence mechanisms ranged against them (1). Vertebrate innate cellular defence pathways include the activation of interferon (IFN)-α and -β and their downstream upregulation of a large number of ISGs in infected and bystander cells with potent antiviral activities. To counter this, a variety of mechanisms have evolved in RNA viruses to evade IFN-mediated cellular defence at all stages of this pathway, targeting recognition and signalling steps and downstream specific evasion or antagonism of individual ISGs. Typically, evasion is mediated by the action of additional virally encoded genes, such as the NS1 of influenza A virus (IAV) (2), through dual purposing of viral proteins that may have unrelated replication functions, such as NS5A of hepatitis C virus (HCV) (3) or finally, through the evolution of additional coding in alternative reading frames, such as the V protein of paramyxoviruses that antagonizes IFN signalling pathways (4).

It is increasingly recognized, however, that virus adaptation to hosts and their successful propagation and onward transmission may determine optimization of more genome-wide attributes of RNA viruses, such as nucleotide composition and internal RNA structuring. RNA base-pairing in single-stranded RNA genomes can indeed create replication and translation elements as well as more pervasive folding associated with RNA virus persistence (5,6). Virus composition also appears to be often tightly constrained within individual virus species or genera. Most strikingly, vertebrate RNA genomes encompass a vast range in G+C contents, as low as 33% in respiratory syncytial virus to 70% in rubella virus, without currently any functional explanation for what drives these compositional differences. Even more striking is the long established observation for the marked suppression of frequencies of CpG and UpA dinucleotides in the genomes of most vertebrate RNA virus groups, and indeed in those of small DNA viruses and retroviruses (7–9). We and others have shown that artificially increasing frequencies of either dinucleotide severely impacts upon the replication abilities of the mutants (10–13). This attenuation mechanism has also been observed, by proxy, in mutants with engineered changes in coding sequences to incorporate disfavoured codon or codon pairs (14–17)—these manipulations had the unintended and unrecognized effect at the time of simply increasing CpG and UpA frequencies (18–20). Using an echovirus 7 (E7) replicon, we have recently shown that equivalent CpG- and UpA-mediated attenuation can be achieved by insertion of additional CpG and UpAs in non-translated regions of the genome (10). Functionally, the attenuation is not mediated through greater activation of IFN or greater susceptibility to downstream ISGs, nor by apoptosis induction, stress response pathways or siRNA (10,11).

Recently, screening of siRNA knockdown libraries identified zinc finger antiviral protein (ZAP) and TRIM25 as proteins that reverse the attenuation of HIV-1 mutants with increased CpG frequencies (21). ZAP was shown to directly bind to CpG motifs in modified RNA sequences and reduce levels of mutated cytoplasmically expressed mRNAs through as yet uncharacterized mechanisms. In the current study, we have investigated the effect of ZAP on the attenuation of virus and replicons of E7 with altered frequencies of CpG and UpA, its binding and downstream effects on translation and RNA stability, both proposed as mechanisms for the antiviral action of ZAP. We further investigated effects of RNAseL on attenuation; activated RNAseL targets single-stranded RNA sequences for cleavage at UpA and UpU dinucleotide sites (22,23), and potentially accounts for the attenuation of viruses with genomes enriched for these dinucleotides—this would include the UpA-high E7 mutants we previously investigated (10,11). RNAseL is activated by 2′–5′ oligoadenylate molecules (2-5A) produced by oligoadenylate synthetase (OAS) 1 and its paralogs, OAS2 and OAS3 (reviewed in (24)). These are interferon-inducible pattern recognition receptors that are triggered to produce 2–5A by binding to double-stranded RNA. As OAS1 and OAS3 have been demonstrated to possess antiviral activity (25–27), we further investigated their role in the attenuation of compositionally altered mutants of E7.

## MATERIALS AND METHODS

### Cells, viruses and reagents

The design and construction of echovirus 7 isolate Wallace infectious clone and replicon was previously described (11). Briefly two regions (R1, R2) in the viral coding sequence were selected for mutagenesis. R1 is 1242 bases in length and extends from VP3 to part of VP1 in the capsid domain. R2 is 1060 bases in length and consists of 3C protease coding sequence. For the replicon, the structural genes of E7 were replaced with a firefly luciferase gene and cloned in pRiboT7 vector. The CDLR control is based around insertion of a shuffled version of R1 and R2 sequences into the E7 virus clone that retains coding and native frequencies of mono- and dinucleotides. Demonstration of its equivalent replication ability to WT virus is essential to verify that any attenuation of dinucleotide modified sequences in these regions did not trivially result from disruption of RNA replication elements or alternative open reading frames in the mutated region.

Cells were maintained in Dulbecco’s modified Eagle medium (DMEM) (Life Technologies) supplemented with 10% heat-inactivated foetal calf serum (FCS) (Gibco), 100 U/ml penicillin and 100 μg/ml streptomycin (Life Technologies). A549 ZAP knockout (k/o) for all three alleles (-/-/-), RNAseL k/o, OAS1 k/o and OAS3 k/o cells, were grown as described with the addition of 2  μg/ml puromycin (Gibco). Echovirus 7 was propagated in rhabdomyosarcoma (RD) cells. Infectivity assays were performed in a 96-well plate serial dilution format using RD cells to determine TCID_50_ / ml. SB216763, guanidinium hydrochloride (GnHCl) and C16 were obtained from Sigma.

### CRISPR/Cas9 Knockout

Double-stranded gRNAs corresponding to the target sequences for ZAP, RNASeL, OAS1 and OAS3 were generated by annealing complementary DNA oligos and cloned into the *BsmB*I restriction site of lentiCRISPR v2 (28,29). lentiCRISPR v2 was a gift from Feng Zhang (Addgene plasmid #52961; http://n2t.net/addgene:52961; RRID:Addgene_52961) and kindly provided to us by Paul Klenerman (University of Oxford, UK). lentiCRISPR v2 constructs containing gRNAs or a random insert instead of gRNA were co-transfected with p8.91 and pVSV-G [both generous gifts from Brian Willet (MRC – University of Glasgow Centre for Virus Research, UK)] into HEK-293T cells using Lipofectamine 2000 (ThermoFisher Scientific) according to manufacturer’s instructions. Forty-eight hours post-transfection (p.t.), the cell medium was ultra-filtrated (0.22 μm filter, Millipore) and the viral suspension used to transduce A549 and RD cells. The transduced cells were selected with puromycin (2 μg/ml) for at least 1 week and then sorted by flow cytometry in a 96-well plate such that each well contained a single cell. Single cell clones were cultured until colonies were visible and subsequently propagated. Western blot analysis as described below was carried out to determine levels of ZAP, RNAseL, OAS1 and OAS3 expression. For the selected clones, genomic DNA was extracted (Qiagen), the cDNA sequence of the gRNA target sites was amplified by PCR (MyTaq, Bioline) using primers listed in Supplementary Table S1 and inserted in a pGEM-T *easy* plasmid (Promega), cultured in *Escherichia coli* JM109 (Promega) and 12 clones were picked at random, cultured and plasmid extracted by miniprep (Qiagen). The sequences served to confirm the disruption of the coding sequence for each cell line tested; where possible two confirmed clonal cell lines were selected for further experiments.

### Replication phenotype of E7 and altered variants

A549, RD and k/o cells were seeded in 6-well plates at 5 × 10^5^ cells per well and infected with E7 wild-type or CpG-H and UpA-H mutants at MOI of 0.01 per cell for 1 h. The inoculum was then discarded, cells were washed with phosphate-buffered saline (PBS) and recovered with 2 ml of 2.5% FCS culture media. At 1, 12, 24, 36, 48, 60 and 72 h post-infection (p.i.) an aliquot of the supernatant was withdrawn and the virus titre determined by TCID_50_ in RD cells. This was performed in duplicate for each virus and each cell line and for two clonal cell lines for ZAP k/o and further cell lines with RNAseL, OAS1 and OAS3 k/o. RNA for each sample was isolated from the viral supernatant, pre-treated with RNase One and extracted with the NucliSENS EasyMAG (Biomerieux). This allowed for quantification of E7 RNA sequences by quantitative real-time PCR; primer sequences are listed in Supplementary Table S1.

### Replicon assay—RNA preparation

Replicon plasmids comprised the Wallace clone of E7 with the structural gene block replaced by a luciferase gene with zero CpGs and lowered UpA frequencies (cu). The replicon was further modified by insertion of modified sequences in the non-translated 3′UTR as previously described (10). Further constructs were made by replacement of the cu luciferase sequence with separate CpG-zero (c), UpA-low (u), codon-optimized (CO) or wild-type luciferase gene sequences using the *Kas*I and *San*DI restriction sites.

The plasmids were linearized using *Not*I, RNA transcripts synthesized *in vitro* with the T7 RNA polymerase kit (Megascript T7, ThermoFisher Scientific) for 4 h. RNA integrity was confirmed by gel electrophoresis before use. pTK-Ren (Promega) expressing renilla luciferase was co-transfected as a transfection control. PTK-Ren was linearized with *Xba*I. The transcripts were DNase treated (RQ1 DNAse, Promega, 1 h at 37°C) and cleaned with a RNA Clean and Concentrator column (Zymo Research). The quantity of RNA was assessed by Qubit fluorometric quantitation (ThermoFisher Scientific).

Cells were seeded at 2 × 10^4^ cells per well in 96-well plates and transfected with 50 ng of replicon and 10 ng of pTK-Ren using MessengerMax transfection reagent (ThermoFisher Scientific). The cells were then incubated for an indicated period of time of 1, 4 or 6 h. The cells were washed with PBS, harvested in passive lysis buffer (Promega) and the luciferase activities were measured using the Dual Luciferase reagent kit and GloMax multi detection system (Promega). To inhibit replication of the replicon, cells were pre-incubated for 2 h with the 10 μM of the replication inhibitor guanidinium hydrochloride (GnHCl) before the transfection step.

The firefly luciferase readings were normalized to the renilla value for each sample. In some formats investigating effects of gene k/o, luciferase expression was further normalized to that of parental A549 cell line control.

### SDS-PAGE and immunoblotting

Samples were resolved on 4%, 7.5% or 12% SDS-PAGE and transferred to a nitrocellulose membrane using a semi-dry transfer unit (Bio-Rad). The membrane was incubated in blocking solution PBS-0.1% tween and 5% skimmed milk (Sigma) and immunoblotted with the following primary antibodies: FLAG (Sigma), RNAseL (Abcam), ZCCHV (for ZAP) (Abcam ab154680), OAS1 (Abcam ab86343) and OAS3 (Abcam ab154270). Antibody binding was detected by HRP-conjugated secondary antibodies followed by chemiluminescence detection by ECL prime western blotting reagent (Ge Healthcare). Images were analysed using Image software, and quantitative data were obtained for three independent experiments for ZAP upregulation.

### *In vitro* translation

The replicons in this study were tested for their translation efficiency in cell-free systems, which contained only the minimum components necessary for translation. Rabbit reticulocyte lysate (Promega) was programmed with 2 μg of transcript RNA prepared as described above. The cell-free system was assembled as follows: 70 μl of cell-free lysate, 2 μl of AA-Met, 2 μl of AA-Leu, 2 μl of RNAsin, 2 μg of RNA transcript, water to a final volume of 100 μl. The mix was vortexed gently and aliquoted into nine tubes, and incubated at 30°C. At various time points (typically 30, 60 and 90 min), three replicates were withdrawn and the luciferase activity read as described in the previous section. This experiment was repeated in wheat germ extract (Promega) programmed with a total of 10 μg of transcript RNA and 2.5 mM potassium acetate.

### Quantitative real-time PCR (qRT-PCR)

mRNA abundance and induction of ISGs were determined by an internally controlled quantitative real-time PCR using primers listed in Supplementary Table S1. Total cellular RNA was isolated using RNeasy kit (Qiagen). About 40 ng RNA was used as the template for qRT-PCR using Superscript III (Invitrogen) and Fast SYBR green Master Mix (Applied Biosystems). The abundance of the target mRNA was normalized to that of HPRT1 housekeeping gene co-amplified by qPCR (primers listed in Supplementary Table S1).

To quantify viral RNA copy numbers, total RNA was extracted from cell supernatants by NucliSENS EasyMAG (Biomerieux). The amount of viral RNA for each time point was determined against a standard curve of quantified transcript RNAs using the Quantitect RNA kit (Qiagen) and primers annealing specifically to the 5′UTR of E7 (Supplementary Table S1). qPCR reactions were performed using a StepOne plus Real time PCR system (Applied Biosystems).

### ZAP phosphorylation

A549 cells were treated with calf Intestine alkaline phosphatase (CIAP, New England Biolabs) and incubated at 37°C for 30 min before being lysed in SDS loading buffer and electrophoresis. ZAP-S was detected by WB using ZC3HAVI antibody (Proteintech) and mobility compared with an untreated control. For investigation of effects of kinase inhibitors on ZAP phosphorylation, A549 cells were treated with 2 μM C16 (Sigma), 5 μM GSK3β inhibitor SB216763 (Sigma) or DMSO for 3 h. Cells were washed with PBS before being lysed in SDS loading buffer. To investigate effects of phosphorylation inhibitors on virus replication, A549 cells were similarly pre-treated with 2 μM C16, 5 μM SB216763 or DMSO for 3 h, prior to infection at an MOI of 10. Supernatant harvested at 24 h was assayed for infectivity in A549 cells as described above.

### Phosphorylation detection

Samples were mixed with 2× SDS loading buffer containing 10% β-mercaptoethanol and heated to 95°C for 10 min. Samples were loaded onto a SDS-8% PAGE gels. Proteins were size separated by electrophoresis and semi-dry blotted onto Immobilin-P membranes (Merck Millipore). The membranes were stained for tubulin (1:2000 in 1% milk powder-PBS-Tween; A11126; Molecular Probes) and human ZAP (1:5000 in 1% milk powder-PBS-Tween; ab154680, Abcam). Finally, the membranes were stained with secondary alkaline phosphatase-conjugated antibodies (α-mouse, A5153, Sigma-Aldrich; α-rabbit, D0487, Dako) and developed with nitroblue tetrazolium (NBT)/BCIP (5-bromo-4-chloro-3-indolylphosphate) (Roche).

### Virus infectivity assay

RD cells were pre-treated with either 2 μM C16, 5 μM GSK3β inhibitor SB 216763 or DMSO for 24 h. Cells were detached from their culture plates and used in a virus infectivity assay by end-point dilution. Infectivity of pre-titred virus stock supernatant was determined for the indicated mutant E7 viruses in the presence of C16, GSK3β inhibitor SB 216763 or DMSO.

### RNA fluorescent *in situ* hybridization (FISH)

Custom Stellaris fluorescent *in situ* hybridization (FISH) Probes were designed against an unaltered WT portion of the E7 genomic RNA (nt 3200–4200) by utilizing the Stellaris RNA FISH Probe Designer (Biosearch Technologies, Inc., Petaluma, CA) available online at www.biosearchtech.com/stellarisdesigner. Probes and detailed staining procedure have been described previously (10). In short, A549 WT cells, ZAP (clone B8) or OAS3 (clone B1) k/o cells were infected at MOI of 5 per cell of the indicated E7 mutant viruses. Cells at 6 hours post-infection were washed with PBS and fixed with 3.7% paraformaldehyde in PBS for 10 min and incubated with primary antibodies against ZAP (1:150, Proteintech) before being stained for E7 RNA using the RNA FISH probe set as previously described (10). Nuclei were counterstained with 4′,6′-diamidino-2-phenylindole dihydrochloride (DAPI). Confocal microscopy images were acquired on a Zeiss LSM880 upright microscope with Airyscan; postacquisition analysis was conducted using Zen software (Zen version 2015 black edition 2.3; Zeiss) or Fiji (version 1.49) software (30).

Cytoplasmic co-localization of E7 RNA with ZAP was estimated through the Pearson’s correlation coefficient (*R*) by PSC Colocalization plug-in (ImageJ-NIH; (31)). *R* ranges between −1 (perfect negative correlation) to +1 (perfect positive correlation) with 0 meaning no correlation. Co-localization calculations were performed on >4 cells from at least two independent experiments.

### Expression of ZAP and RNAseL in different cell types

Cells in 24-well plates were either pre-treated with 50 ng/ml human interferon-β (Calbiochem) for 24 h or untreated controls before being lysed in passive lysis buffer (Promega). RNA was isolated and pRT-PCR reactions were performed using a StepOne plus Real time PCR system (Applied Biosystems) as described above.

### RNA-binding protein immunoprecipitation

RNA-protein immunoprecipitations (RIP) were carried out using the Magna RIP kit (Millipore). Cells were seeded in 15 cm dishes and transfected with 10 μg of Region 2 viral RNAs: WT, CpG High R2 or UpA High R2. In parallel, two dishes were mock transfected without viruses. After 5 h of incubation, the media were discarded and the cells washed with cold PBS twice. The cells were scraped in 10 ml of cold PBS and harvested by centrifugation at 1500 rpm, for 5 min, then resuspended in an equal volume of cell pellet in RIP lysis buffer containing protease and RNAse inhibitors. Two aliquots of 10 μl each were stored as inputs for all samples.

Magnetic beads protein A/G were associated with 5 μg/ sample of Rabbit anti-ZAP (Proteintech) or normal Rabbit IgG (Millipore) as negative control. Cell lysates were incubated for 5 h and the samples were washed six times, with complete resuspension of beads between washes and 15 min incubation with rotation for the last two washes. After washings, the samples were re-suspended in RIP wash buffer and divided in aliquots. A 100 μl aliquot of immune-complexes was reserved for analysis of the protein fraction. After beads were immobilized, the wash media were discarded and beads were re-suspended in Laemmli sample buffer. About 10 μl of the inputs were also mixed with Laemmli buffer. All the samples were heated for 5 min at 95°C and separated by SDS-PAGE for western blot analysis. In parallel, viral RNA were purified from another aliquot of immune-complexes and inputs, using the RNA-viral kit (Zymo research). RNAs were eluted in 15 μl of RNase–DNase free water. The copy number of E7 present in each fraction was determined by qPCR as described above.

### Reconstitution of ZAP expression

About 5 μg of ZAPeGFP plasmid was transfected into B8 ZAP k/o cells cultured in 10 cm dishes. After 16 h of expression, the cells were transfected with 5 μg of WT, CpG-high or UpA-high 3′UTR replicons and 500 ng of TKren RNAs. The cells were incubated for 6 h, then trypsinised and harvested in 1% FCS-PBS buffer and sorted for green fluorescence using a BDFACS ARIAIII sorter to isolate ZAP-expressing cells. About 30 000 cells / well in a 96wp were harvested in 50 μl passive lysis buffer and assayed for firefly and renilla luciferase expression as described above.

### Bioinformatics analysis

Compositional modification of dinucleotide frequencies in E7 and luciferase sequences was performed previously (10,11) using the program Sequence Mutate in the SSE package (32). For compositional analysis of cellular mRNAs, non-redundant human mRNA sequences were downloaded from the http://www.ncbi.nlm.nih.gov/gene database, with sequences shorter than 250 bases excluded. The Composition Scan program in the SSE v. 1.3 package was used to determine dinucleotide frequencies and metrics of codon use. Codon adaptation indices were calculated using the E-CAI server (33).

## RESULTS

### Effects of ZAP expression on E7 replication

**Figure 1.**
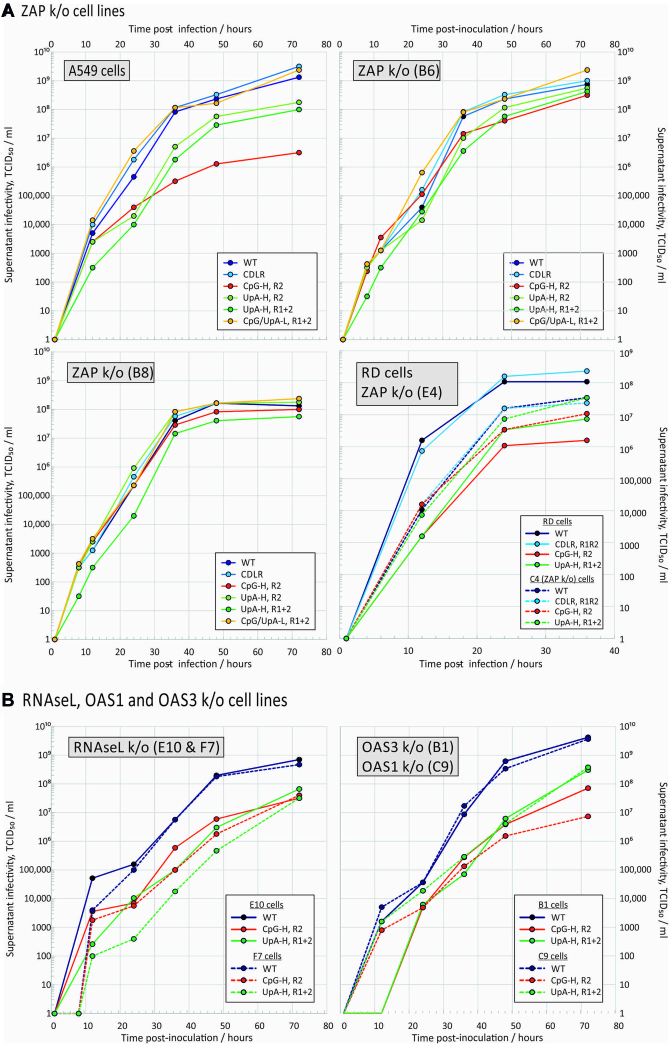
Reversal of CpG and UpA-mediated attenuation in k/o cells. Multi-step replication kinetics of E7 WT and compositionally altered mutant viruses in A549 and RD cells, and in cell lines with targeted knockout of (**A**) ZAP and (**B**) RNAseL, OAS3 and OAS1. Cells were infected with a MOI of 0.01 and supernatant sampled and titred for infectivity at different time points after infection. Graph lines show mean TCID_50_ values of supernatants from two biological replicates.

Replication rates of E7 and a series of mutant viruses with modified composition in R1 and R2 coding regions were compared in WT A549 cells and two separately CRISPR-derived ZAP k/o cell lines, B6 and B8 with verified knockout of ZAP expression (Figure 1A and Supplementary Figure S1). In A549 cells, E7 mutants with increased frequencies of CpG dinucleotides in the R2 region showed substantially attenuated replication, with a 3 log reduction in supernatant TCID_50_s compared to those of the WT virus at 72 h (Figure 1A), consistent with previous observations (11). Similarly, the UpA-H R1R2 mutant showed a 1.5 log reduction in end point infectivity compared to WT. The attenuation of CpG- and UpA-high mutants was reproduced in the RD cell line. Replication of the CDLR control E7 mutant was comparable to that of the WT virus (Figure 1), as was the cu mutant of E7 with minimized frequencies of CpG and UpA in R1 and R2. The equivalent replication of the CDLR mutant show that sequence permutation without increasing CpG or UpA dinucleotide frequencies had no damaging effect on virus replication

In the two A549-derived ZAP k/o cell lines, B6 and B8, and in the RD ZAP k/o cell line, E4, the attenuation shown by the CpG-H and UpA-H mutants was almost entirely reversed, with replication kinetics comparable to that of the WT and CDLR control throughout the time course of the experiment (Figure 1A and Supplementary Figure S2). The same close to complete reversion of the attenuated phenotype was observed in the B8 cell line in which replication was monitored by qPCR for E7 RNA (Supplementary Figure S3). In this case, it was additionally possible to demonstrate the close to complete reversion of a CpG-high R1R2 mutant that was too attenuated in A549 cells to accurately titrate in infectivity assays.

**Figure 2.**
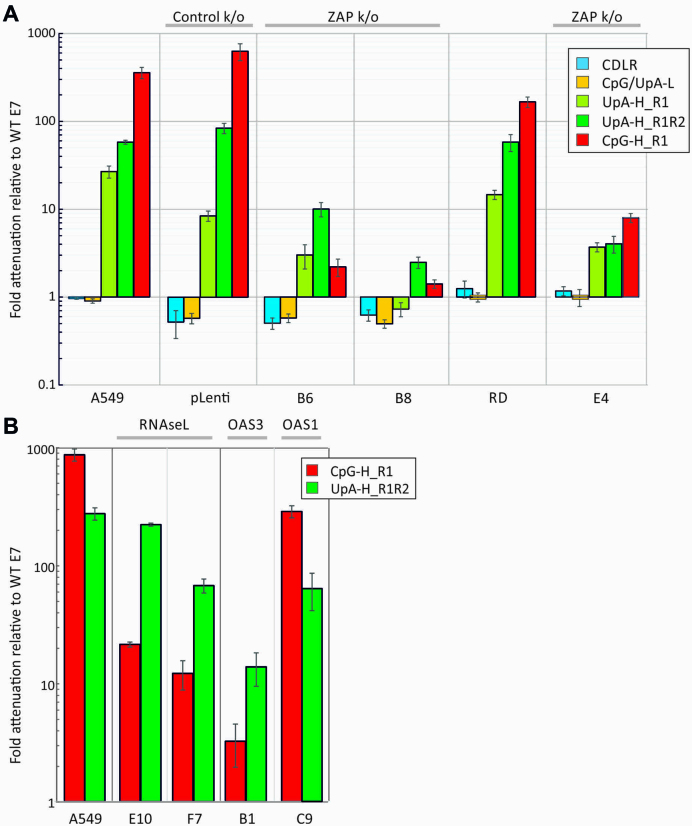
Relative attenuation of compositionally altered mutants of E7 in A549s and ZAP, RNAseL, OAS1 and OAS3 k/o cell lines. Attenuation expressed as the ratio of virus replication of mutant viruses to that of WT virus in (**A**) A549 and two ZAP k/o cell lines (B6, B8); RD and ZAP k/o E4 and (**B**) RNAseL, OAS1 and OAS3 k/o cell lines (E10 and F7, E4 and B1, respectively). Infectivity titres were determined at 36 h post-infection (24 h for RD cells). Bar heights represent the mean of three biological replicates; error bars show standard errors of the mean.

To determine more accurately the extent of replication differences between A549 and RD ZAP k/o cell lines, infectivity titres were determined for three biological replicates at 36 h post-infection during the exponential phase of replication (24 h in RD cells), with values from mutant viruses normalized to those of WT virus in the same cell line (Figure 2A). The CpG/UpA-L and CDLR control variants were unaffected by ZAP expression differences, while there was a large change in the attenuation of the CpG-H high mutant from its >300-fold reduced replication in A549 cells to around a 1.5–2.5-fold reduction in B6 and B8 k/o cells. The attenuation of the UpA-high mutants similarly almost entirely reversed in both ZAP k/o cell lines. As a control for the CRISPR mutagenesis of cells, replication of WT and E7 mutants in the pLenti CRISPR cell line was comparable to that observed in the parental A549 cell line.

**Figure 3.**
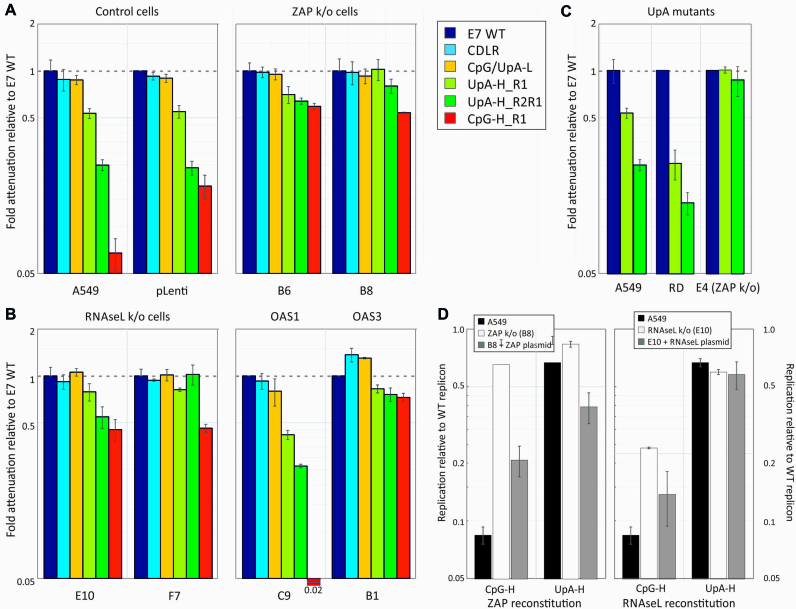
Replication of replicons with 3′UTRs of different dinucleotide compositions compared to WT virus. (**A**–**C**) Effect of pathway knockout on the attenuation of replicons with different CpG and UpA compositions, scored by luciferase expression at 6 h post-transfection. Replicons were modified through insertion of mutated R1 sequences in the non-translated 3′UTR. The UpA-H R2R1 mutant possessed a further insertion of UpA-high sequence in the R2 (coding) region. (A) Attenuation of replicons relative to E7 WT in the parental A549 cell line, a control CRISPR k/o cell line (pLenti) and two ZAP k/o cell lines, B6 and B8. (B) Attenuation in two different RNAseL k/o cell lines and those with k/o of OAS1 and OAS3. (C) Attenuation of UpA-H mutants in A549, RD and E4, an RD-derived cell line with ZAP k/o. Bar heights represent the mean of three biological replicates; error bars show standard deviations. (**D**) Effect of reconstitution of ZAP and RNAseL on replicon replication in the corresponding k/o cell lines (B8 – ZAP and E10: RNaseL). These were transfected with plasmids expressing ZAP or RNAseL with a fused GFP tag to enable FACS-based cell sorting for expression prior to transfection with WT and CpG- and UpA-high replicons at 15 h. Luciferase expression was subsequently recorded at 6 h post transfection, and normalized to expression with WT virus. Expression in A549 and the corresponding k/o cell lines were determined as controls. Bars represent the mean of two biological replicates, each with three technical replicates, error bars show standard deviations.

To further investigate the effects of ZAP on CpG- and UpA-high modified sequences, we assayed previously described E7 replicons with modifications to the 3′UTR that incorporated non-translated sequences of different dinucleotide compositions (10). Any attenuation observed in this replicon is thus independent of effects of CpG and UpA modification of codon usage and downstream possible effects on translation efficiency (10). The 3′UTR insertions comprised sections of the E7 genome (R1) corresponding to the WT virus, sequences modified by CDLR, and further mutants with elevated or reduced frequencies of CpG and/or UpA dinucleotides (10). The replicon with the high CpG R1 3′UTR sequence showed ∼10-fold reduced replication compared to the WT control, which was almost completely reversed in the B8 ZAP k/o cell line (Figure 3A).

The degree of replication attenuation observed in the UpA-high 3′UTR mutant was more limited (60% of WT levels), consistent with previous data (10). To more robustly investigate UpA-induced attenuation in the replicon system, we constructed a further UpA-high R2R1 mutant with a UpA-high region also inserted into the R2 coding region. This created a genomic sequence with 212 additional UpA dinucleotides instead of 109 (11), and consequently showed a greater degree of attenuation in A549 and RD cells (25% and 12% of WT levels). Both the original UpA-high R1 and more attenuated R2R1 mutant reverted to WT replication levels in A549-derived B6 and B8 k/o cells and in equivalent RD k/o, E4 (Figure 3A and C). No reversion of UpA- or CpG-induced attenuation was observed in the pLenti CRISPR control cell line (Figure 3A)

### RNAseL- and OAS3-mediated attenuation

RNAseL activated by 2–5A produced by OAS1, 2 and 3 on dsRNA recognition cleaves single-stranded RNA sequences at UpA and UpU dinucleotide sites. This represents an additional or alternative potential pathway mediating the attenuation of compositionally modified mutants. We generated further k/o cell lines with verified ablated RNAseL genes (Supplementary Figure S1). Even though complete reversion of CpG- and UpA-induced attenuated phenotypes was observed in ZAP k/o cells, partial reversion of the attenuation of both CpG-high R2 and UpA-high R1R2 mutant viruses was also observed in these further k/o cell lines (Figures 1B and 2B) and in the corresponding replicon mutants (Figure 3B).

To investigate the activation pathway for RNAseL and downstream effects on dinucleotide-induced attenuation, we additionally made knockout A549 cell lines with deletions of OAS1 and OAS3 (Supplementary Figure S1). Culture of E7 and mutants in the OAS1 k/o cell line led to replication kinetics of the WT, CpG-H and UpA-H mutant viruses and replicons comparable to that of the parental cell line (Figures 1B, 2B and 3B), with the exception of the CpG R2 mutant, whose replication was actually more attenuated than it was in the parental A549 cell line. Contrastingly, both CpG- and UpA-associated attenuation was largely or entirely reversed in the OAS3 k/o cells (C9).

To rule out unintended effects of CRISPR k/o of ZAP on other cellular pathways, we reconstituted ZAP and RNAseL expression from ZAP-L / eGFP co-expressing plasmid and compared the degree of attenuation of CpG-high and UpA-high 3′UTR mutants of the E7 replicon with those observed in corresponding k/o cells and in the parental A549 cell line (Figure 3D). For ZAP, replication of the high CpG mutant was attenuated in A549 cells, replicated similarly to WT replicon in B8 k/o cells but showed renewed attenuation in cells transfected with the ZAP containing plasmid (Figure 3D, left). The modest attenuation of the UpA-high single region mutant in A549 cells was similarly reversed in B8 cells and but then greatly enhanced in the ZAP-plasmid expressing cells. Comparable effects of RNAseL reconstitution were also observed, with attenuation of the CpG-high replicon similar in A549 cells and the k/o cell line with plasmid-derived RNAseL expression (Figure 3D, right), while there was partial reversion of CpG-induced attenuation in RNAseL k/o cells.

### Binding of CpG and UpA enriched RNA sequences to ZAP

**Figure 4.**
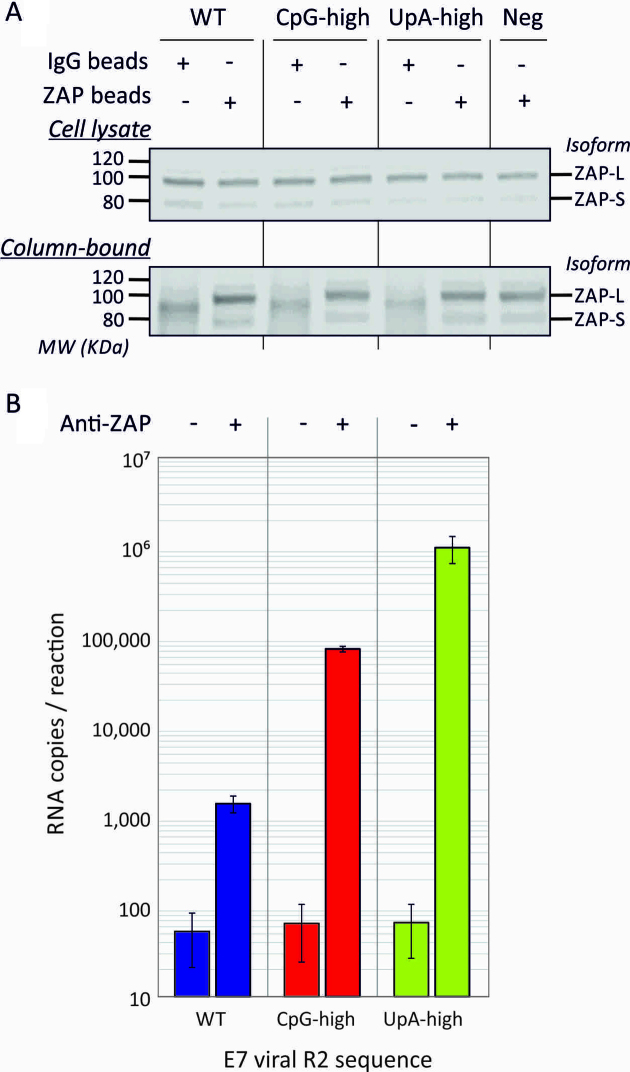
Binding of affinity isolated ZAP to CpG-H and UpA-H RNA transcripts. (**A**) ZAP detection by WB in input A549 cell lysates before and after immunoprecipitation (IP) with anti-ZAP or irrelevant antibody control (lower panel; IP). Long and short isoforms of ZAP migrated at around 90 and 75 KDa, respectively. The negative control was mock transfected (no viral RNA control). (**B**) Quantitation of E7 viral RNA transcripts with WT, CpG-high and UpA-high R2 sequences by qPCR in immunoprecipitated ZAP (right column) or mock precipitated control (left column). Bar heights show the mean of two biological experiments, errors bars show standard errors of the mean. The no RNA transcript control was negative by qPCR (data not shown).

Specific binding of ZAP to CpG-high RNA has been previously demonstrated by a cross-linking-immunoprecipitation assay (21). In the current study, we investigated whether the attenuation of UpA and its reversal in ZAP k/o cells was also associated with ZAP binding by immunoprecipitation of ZAP from A549 lysate and measurement of its binding to RNA transcripts of WT, CpG-high and UpA-high R2 mutants (Figure 4A). ZAP expression was readily detectable in untreated A549 cell lysate (upper panel) and specifically retained by ZAP-specific antibody after immunoprecipitation (IP; lower panel). Addition of RNA transcripts followed by stringent washing revealed marked differences in the relative amounts of RNA bound to the immobilized ZAP (Figure 4B), with minimal binding of WT sequence but ∼100-fold greater retention of CpG-high viral RNA and 1000-fold greater retention of UpA-high viral RNA. No binding of any of the RNA transcripts was observed in columns containing a rabbit negative control antibody (mean 46 copies, SEM ±33).

### Effect of ZAP, RNAseL and OAS3 k/o on E7 entry

We previously showed that restriction in the replication of CpG and UpA-high mutants of E7 was mediated early after infection—mutants were able to enter cells and initiate translation but these subsequently failed to progress to the formation of replication complexes and the generation of infectious progeny viruses. To investigate whether ZAP or the RNAseL/OAS3 pathway was mediating this restriction, we infected A549, B8 (ZAP k/o), E10 (RNAseL k/o) and B1 (OAS3 k/o) cells at an MOI of 10 with E7 WT and single or double region CpG or UpA modified viruses. Infection was quantified through fluorescent *in situ* hybridization (FISH) for E7 RNA sequences.

**Figure 5.**
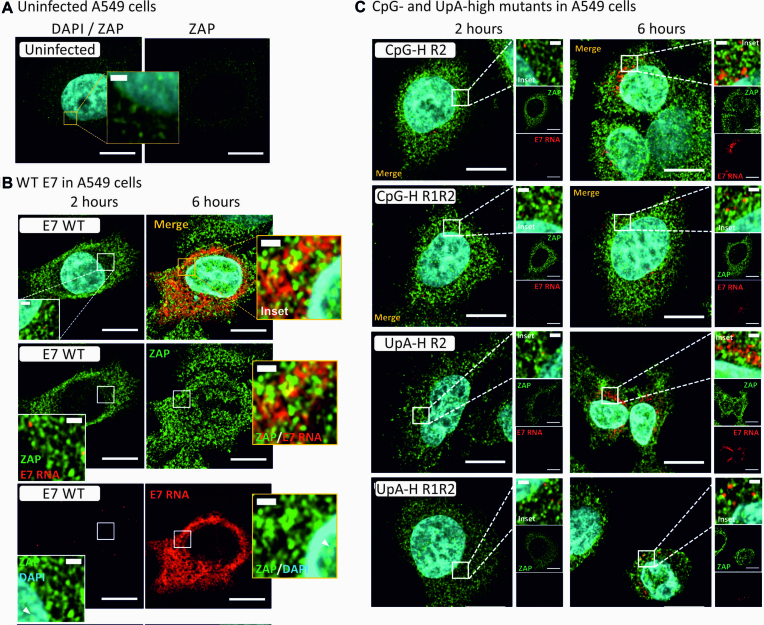
E7 entry and replication initiation in A549 cells. (**A**) Uninfected A549 cells co-stained for ZAP by specific antibody and GFP-coupled second antibody and for E7 RNA by FISH (assay specificity control, not detected). Nuclear DNA was stained by DAPI (blue). (**B**) E7 RNA detection by FISH in cells at 2 and 6 h post after infection with WT E7 co-stained for ZAP. (**C**) Merged images of A549 cells infected with compositionally modified mutants of E7, CpG-high in R2 or R1 and R2 combined, UpA-high in R1 or R1R2.

Infection of A549 cells with E7 WT led to the rapid appearance of detectable E7 RNA sequences by FISH from a few punctate FISH signals likely representing early established replication complexes at 2 h and the subsequent spread of E7 through the entire cytoplasmic space by 6 h (Figure 5B). Low level cytoplasmic expression of ZAP was detected in uninfected cells (Figure 5A), and this increased substantially at 2 and 6 h post-infection with WT virus, associated with a prominent relocation of ZAP signals into nucleolar compartments in the nucleus at the later time point (Figure 5B). E7 RNA and ZAP did not substantially co-localize in infected A549 cells, with largely separate red and green foci evident at the 2 h time point, and a co-localization coefficient of 0.57 (Supplementary Figure S4 and Supplementary Table S3).

The replication of CpG- and UpA-high mutants was highly restricted at 2 and 6 h time points compared to E7 WT for all mutants (Figure 5C). Replication of both UpA- and CpG-high mutants was largely confined to the perinuclear cytoplasm. Infected cells also showed a similar upregulation of ZAP expression and nuclear relocalization but again with incomplete co-localization with E7 replication sites. However, calculated coefficients of co-localization were significantly higher in the CpG- and UpA double region (R1R2) mutants compared to E7 WT RNA (Supplementary Table S3), consistent with their potential greater interaction with ZAP than WT virus.

**Figure 6.**
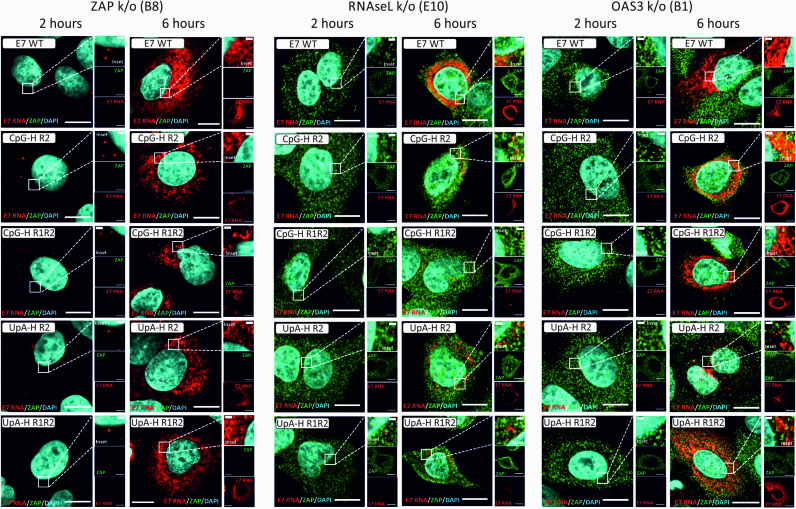
Replication of E7 WT and compositionally altered mutants in different k/o cell lines Merged images (E7 RNA, ZAP and nuclear DNA) of E7 WT and UpA- or CpG-high mutants of E7 in ZAP, RNASeL and OAS3 k/o cell lines.

Closely mirroring the results from the virus infectivity and replicon assays (Figures 1–3), the attenuated replication of UpA- and CpG-high mutants was partly (RNAseL k/o) or entirely reversed (ZAP and OAS3-k/o cell lines, Figure 6). Infections in the RNAseL k/o cells were again largely confined to perinuclear regions of the cytoplasm, while E7 RNA FISH signals developed throughout the cytoplasm between the 2 and 6 h time points in the ZAP and OAS3 k/o cells.

**Figure 7.**
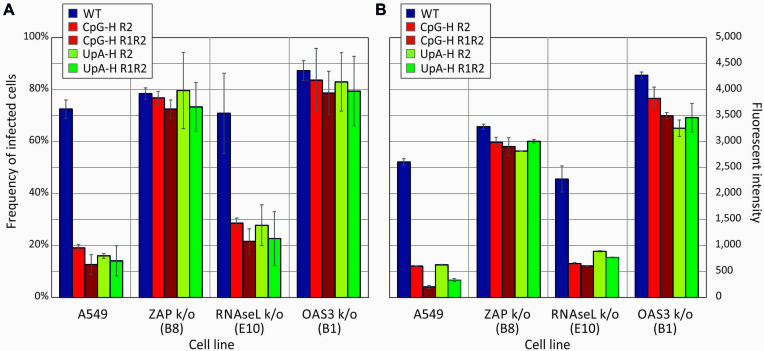
Frequencies and fluorescent intensities of infected cells detected by FISH. (**A**) Quantitation of FISH-positive cell frequencies by Airyscan post-acquisition analysis in representative fields of cell monolayer infected with E7 WT and compositionally modified mutants of E7. (**B**) Quantitation of fluorescent intensity of infected cells. Bar heights show the mean of two biological replicates; error bars show ± 1 standard deviation.

Using post-acquisition analysis of the images (Airyscan), FISH signals in representative sections of each virus/cell combination were quantified for cellular infection frequencies (Figure 7A) and for fluorescent intensities of infected cells (Figure 7B). Frequencies of A549 cells infected by E7 WT of around 70% were substantially higher than the 12–19% frequencies observed with the CpG- and UpA-high mutants (Figure 7A, left). Cells infected with E7 WT were additionally substantially brighter (Figure 7B, left). However, UpA- and CpG-high mutants showed both infected cell frequencies and brightness’s matching that of E7 WT in ZAP and OAS3 k/o cells, consistent with the microscopic appearance in the section depicted in Figure 6. Again, comparable to what was previously observed in cell culture, there was a partial phenotypic reversion of CpG- and UpA-high mutants in RNAseL k/o cells (21–29% infection frequencies, and ∼2-fold increases in fluorescent intensities compared to A549 cells.

### Effects of CpG and UpA modifications on translation efficiency

In addition to effects on virus and replicon replication, CpG and UpA dinucleotides have been hypothesized to influence translation rates of viral templates and modulate replication speed (34,35). The previous findings that CpG and UpA additions to the 3′UTR of E7 had similar effects on replication attenuation as those added in coding region (10) rule out direct effects of compositional modification on translation efficiency. However, ZAP and RNAseL are both multifunctional proteins and their activation may influence translation initiation and processivity through interactions elsewhere on the RNA template. To investigate this potential attenuation mechanism, we determined effects of dinucleotide frequency modifications on translation in a series of constructs and assay formats.

**Figure 8.**
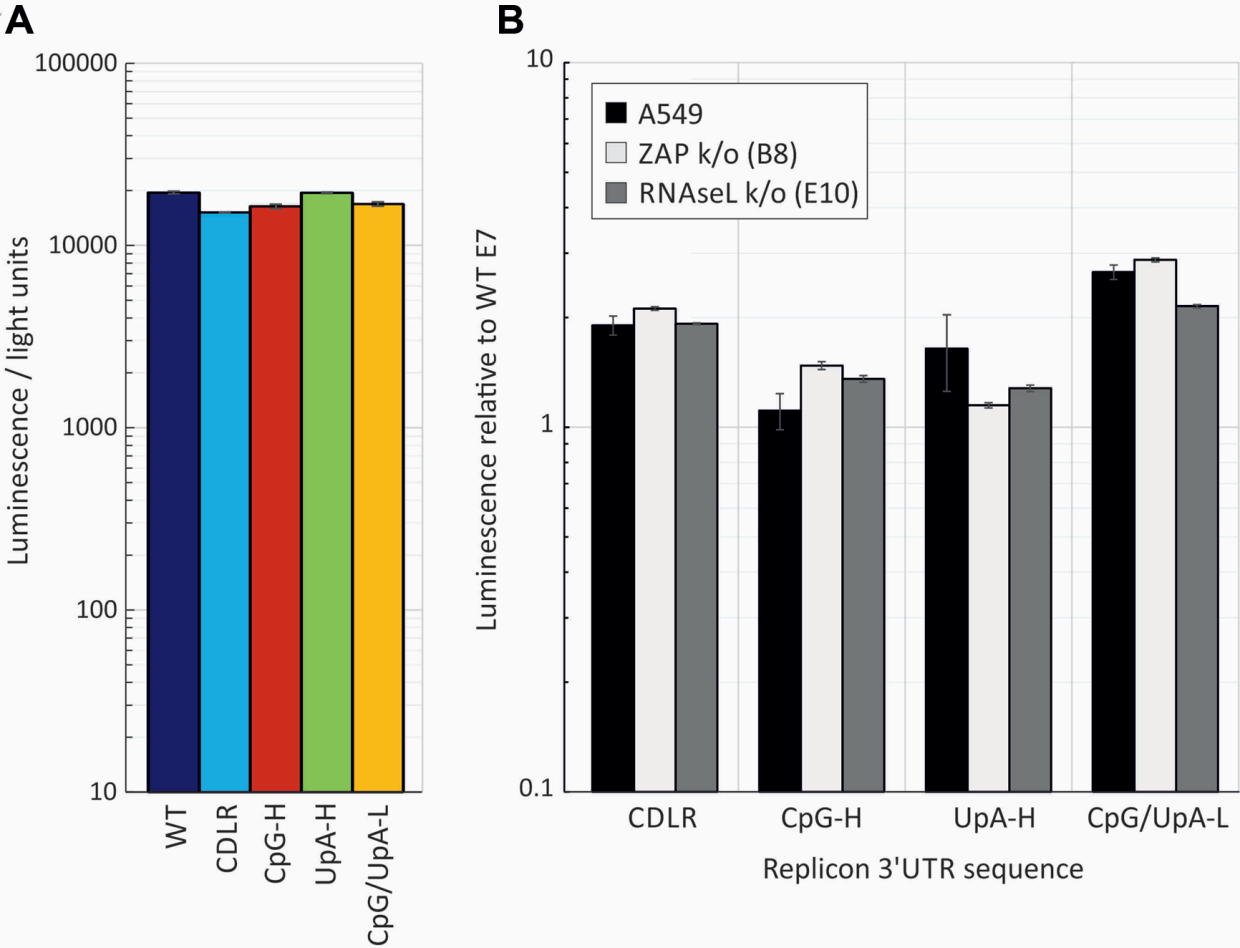
(**A**)Translation rates of replicon templates in the absence of replication Translation of E7 replicons with inserted WT, CDLR, CpG-H, UpA-H, CPG/UpA-low R1 sequences into the 3′UTR in a cell-free translation assay; translation was recorded by luciferase expression at 30 min post-transfection. Histograms show mean values from three technical repeats; error bars show SDs (**B**) A549, A549-ZAP k/o (B8 clone) and RNAseL k/o (E10 clone) cell lines were co-transfected with the same replicons in the presence of 10 μM GnHCl. Renilla luciferase was co-transfected to normalize for differences in transfection efficiency—expression was expressed as the ratio of the two luciferase signals relative to that of the WT replicon control (*y*-axis). Histogram values derive from two biological repeats, each with three technical replicates; error bars show SDs.

**Figure 9.**
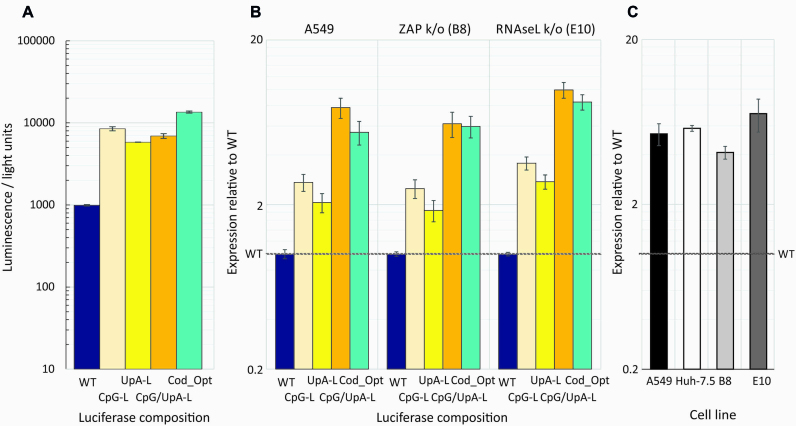
Translation rates of luciferase genes in the E7 replicon. (**A**) Translation of E7 replicons with compositionally modified luciferase genes in a cell-free translation assay; translation was recorded by luciferase expression at 30 min post-initiation. (**B**) Normalized luciferase expression of the same constructs in A549 cells and ZAP and RNAseL k/o cells at 1 h post-transfection with in the presence of 10 μM GnHCl to inhibit replication. Data were derived from three biological replicates; error bars show SDs. (**C**) Fold increase in translation of the CpG/UpA-Low luciferase gene over WT Luc in the HCV replicon in different cell lines in the presence of GnHCl at 3 hs post transfection. Data were derived from three technical replicates; error bars show SDs.

To isolate effects of replication from translation, we assayed translation in cells treated with the replication inhibitor, guanidine hydrochloride (GnHCl) and in cell-free *in vitro* assays in rabbit reticulocyte lysates. Assays compared translation rates of replicons modified by compositional changes to the 3′UTR (Figure 8) or to the luciferase reporter gene at the start of the polyprotein (Figure 9). Luciferase gene modifications comprised removal of CpGs and/or the maximum number of UpA dinucleotides while preserving protein coding (Supplementary Table S1 (11)). A further codon optimized mutant was made to maximize translation rates (Supplementary Table S2).

Replicons with the modified 3′UTRs showed equal translation rates in the cell- free reticulocyte lysate indicating that modifications to the 3′UTR has no effect on translation initiation or processivity through the luciferase gene in a cell-free context (Figure 8A). The 3′UTR modification similarly showed no influence on translation in A549 cells in the presence of 10 μM GnHCl that inhibited virus replication at 1 h post-transfection (Figure 8B) and at 4 and 6 h (data not shown). Translation from the different replicons was unaffected by ZAP and RNAseL k/o.

Modification of the luciferase gene by removal of CpG and/or UpA dinucleotides or by codon optimization showed similar effects on translation in transfected cells as was observed in rabbit reticulocyte and wheat germ extract *in vitro* translation assays (Figure 9A and Supplementary Figure S5). In both, the WT firefly derived luciferase gene showed 5–10-fold lower translation that constructs with modified luciferase genes where CpG or UpA dinucleotides had been removed or the sequence was codon-optimized. Similar, composition-dependent variability in translation was observed when the replicons were transfected into A549 cells in the presence of GnHCl (Figure 9B; left panel), with up to 8-fold increased translation. This composition-associated difference in translation rate was comparable when the constructs were transfected into ZAP and RNAseL k/o cells (Figure 9B; middle and right panels). The previously described enhanced expression of CpG-zero, UpA-low Luc in HCV replicons (36) was similarly independent of ZAP or RNAseL expression (Figure 9C). The modified luciferase gene showed between 4- and 7-fold greater expression than the WT sequence in the original Huh7.5 cells, A549s and both B8 and E10 k/o cell lines in the presence of GnHCl.

In summary, translation rates of the luciferase gene were influenced by CpG and UpA composition as well as by codon optimization both *in vivo* and in cell-free assays, but not influenced by ZAP or RNAseL expression. Compositional changes elsewhere in the E7 replicon (3′UTR) had no effect on translation in either translation assay format.

### RNA degradation by ZAP and RNAseL

**Figure 10.**
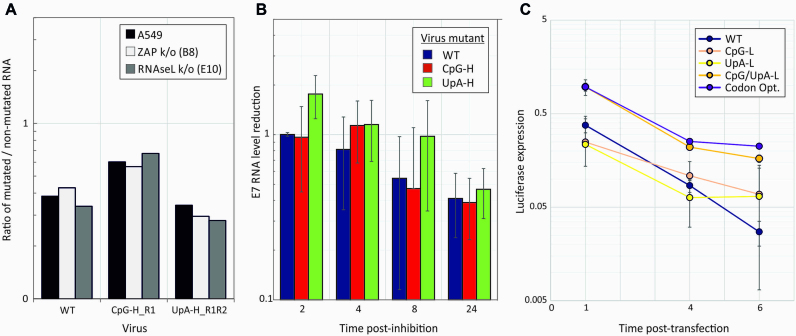
Integrity and stability of compositionally modified E7 genomic RNA. (**A**) RNA was extracted from E7-infected cells and RNA quantified in a non-mutated (5′UTR) and mutated region (R2) by qPCR. Ratios of viral loads between the two regions were normalized to those obtained by the same qPCR of full-length RNA transcripts of WT and each mutant. (**B**) Decay rates of E7 RNA in A549 cells infected with E7 WT, R1R2 UpA-H and R2 CpG-H mutants after replication inhibition by 10 μM GnHCl. E7 RNA was extracted from infected cells at the indicated time points post-inhibition and quantified by qRT-PCR; levels were expressed as ratios to levels of RNA immediately pre-addition of GnHCl. Histogram height represent the mean of three technical replicates; error bars indicate SDs. (**C**) Luciferase expression at 1, 4 and 6 h post transfection of A549 cells with replicons containing compositionally modified luciferase gene sequences in the presence of 10 μM GnHCl to inhibit replication. Points represent the mean of three biological replicates; error bars show 1 SD.

RNAseL is a nuclease that targets UpU and UpA dinucleotides on activation with 2′-5′A (reviewed in (24)). ZAP has been reported to sequester RNA and target it for exosomal degradation (37). Endonuclease or exonuclease degradation of RNA sequences with increased CpG or UpA frequencies may potentially underlie to the replication attenuation and translation reductions of compositionally modified E7 constructs. To investigate this, WT and mutants with modified dinucleotide compositions in R2 were grown in cell culture and the integrity of the viral RNA genomic sequences evaluated by qPCR using primers targeting mutated and non-mutated regions of the genome (Figure 10A).

*C*_t_ values in the qPCR for the (unmodified) 5′UTR were comparable to those of the mutated R2 region for all three RNA transcripts. These ratios were closely reproduced in viral RNA extracted from A549 cells infected with WT, CpG-high and UpA-high mutants (Figure 10A), indicating that all three variants were substantially intact in replicating cells. Ratios were unaffected by ZAP and RNAseL k/o, indicating that neither pathway was involved in specific degradation of mutated virus genome regions.

To investigate whether higher rates of degradation occurred in compositionally abnormal RNA engaged in active virus replication, A549 cells were infected at an MOI of 1 and incubated until an early CPE developed in the WT-infected cells (18 h). Replication was then terminated by addition of 10 μM GnHCl, and viral RNA quantified by qPCR of the 5′UTR region at different time points post addition (Figure 10B). Decay rates of CpG-high and UpA virus mutants were comparable to those of WT E7 over the following 24 h. Decay rates were also comparable in ZAP and E10 k/o cells (data not shown). Similar decay rates of replicons with compositionally modified luciferase gene sequences were observed in GnHCl-treated A549 cells at 1, 4 and 6 h post-transfection (Figure 10C). Decay rates were comparable between replicon constructs in ZAP and RNAseL k/o cells (data not shown).

### Functional dependence of ZAP function on phosphorylation

**Figure 11.**
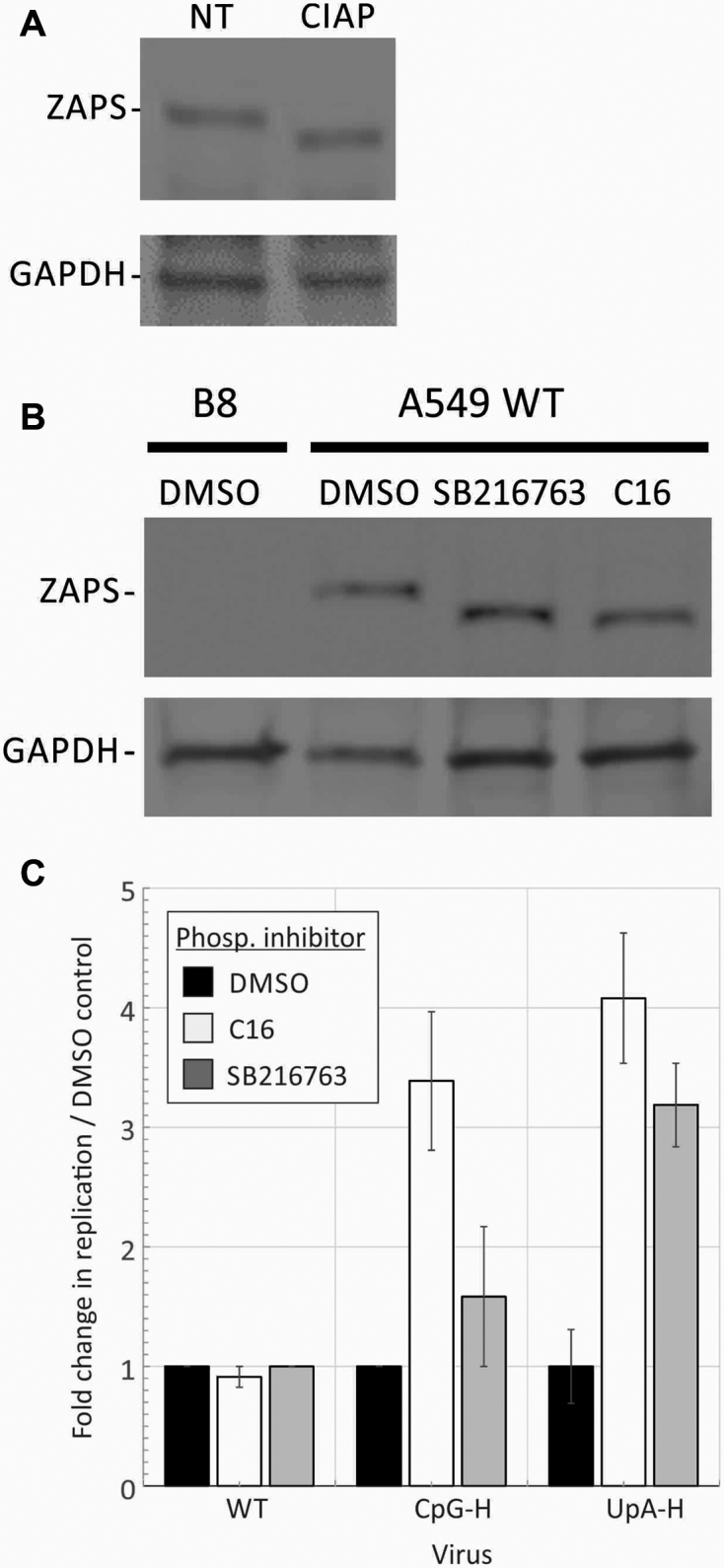
Phosphorylation of ZAP and effects on E7 replication. (**A**) A549 cells were treated with CIAP, lysed and the mobility of ZAP-S identified on WB compared to a non-treated (NT) control. (**B**) A549 WT cells were treated with either C16, the GSK3β inhibitor SB216763 or DMSO (negative control) and mobility compared on WB. B8 cells (left lane) expressed no ZAP-S. (**C**) Comparison of replication titres of R2 CpG-H and R1R2 UpA-H virus mutants with that of WT virus after pre-treatment of A549 cells with C16 and SB216763 or mock-treated with DMSO. Graphs show ratios of infectivity titres at 24 h post-infection to that of the mock-treated WT control. Error bars show standard deviations of two biological repeats.

It has been previously reported that antiviral activity of rat ZAP is dependent on phosphorylation by cellular glycogen synthase kinase 3β (GSK3β) (38). To investigate whether phosphorylation of human ZAP also influenced its antiviral effect against compositionally altered variants of E7, we compared replication rates of WT and CpG-high E7 viruses in cells pre-treated with the GSK3β inhibitor, SB 216763 (Figure 11). Cells were also treated with C16, another kinase inhibitor which we have previously shown led to reversion of CpG-mediated attenuation (11).

Dephosphorylation by CIAP led to a mobility shift of ZAP-S (Figure 11A) that was replicated by treatment with the kinase inhibitors SB216763 and C16 (Figure 11B). Pre-treating cells with either inhibitor before infection with R2 CpG-H or R1R2 UpA-H modified E7 led to substantially increased replication, while neither influenced the replication of WT E7 (Figure 11C). These observations both show that ZAP’s ability to attenuate CpG and UpA high viruses require phosphorylation on ZAP and furthermore provide a functional observation for the previously reported reversal of E7 attenuation by C16 (11).

### Expression of ZAP, RNAseL and OAS3 in different cell types

**Figure 12.**
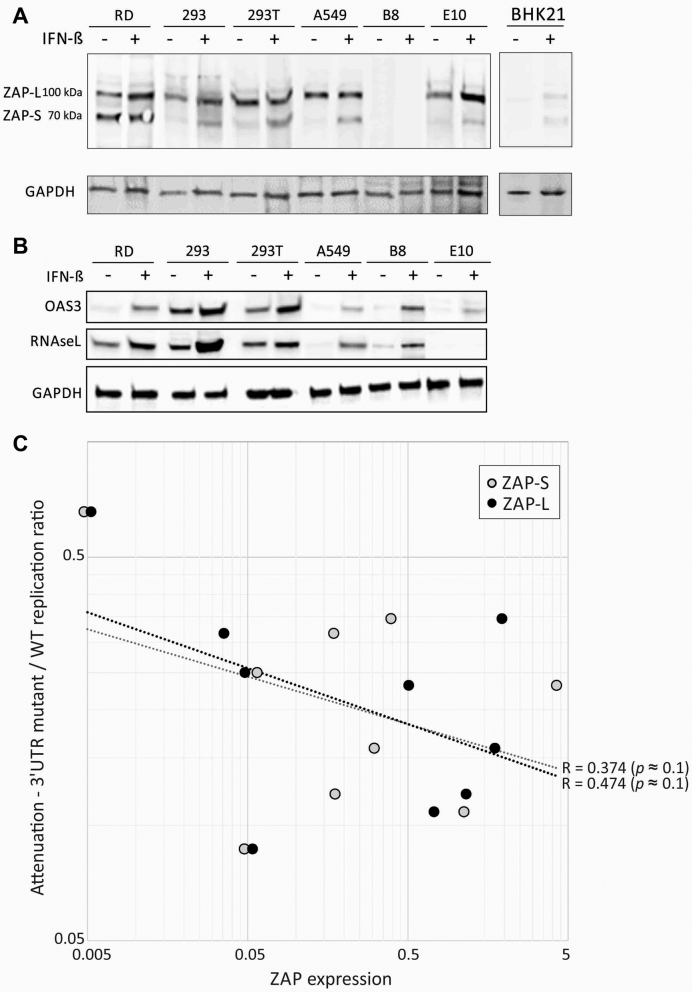
Expression of ZAP, RNAseL and OAS3 in cell lines; association of ZAP expression with attenuation. Detection of ZAP-L and ZAP-S (*A*) and RNAseL, and OAS3 (*B*) by western blot analysis of lysates of different cell lines before and after stimulation with exogenous IFN-β. (**C**) Association of ZAP-L expression with the attenuation index of mutants of E7 with high CpG or UpA 3′UTR sequences. The black and grey dotted lines show lines of best fit and associated *R* values. Significance levels were based on one-tailed probabilities using the Spearman non-parametric test.

The extent to which these recognition and effector pathway-associated proteins might be expressed constitutively and after IFN stimulation was investigated by WB detection in a range of human cell lines (Figure 12A and B). A549 cells showed IFN-inducible expression of RNAseL, OAS3 and the short isoform of ZAP (ZAP-S). Contrastingly, ZAP-L was expressed constitutively. RD and HEK-293 cells showed more extensive constitutive expression of ZAP(-L) and RNAseL; the two HEK93 cell lines furthermore showed high level expression of OAS3 without IFN stimulation.

**Figure 13.**
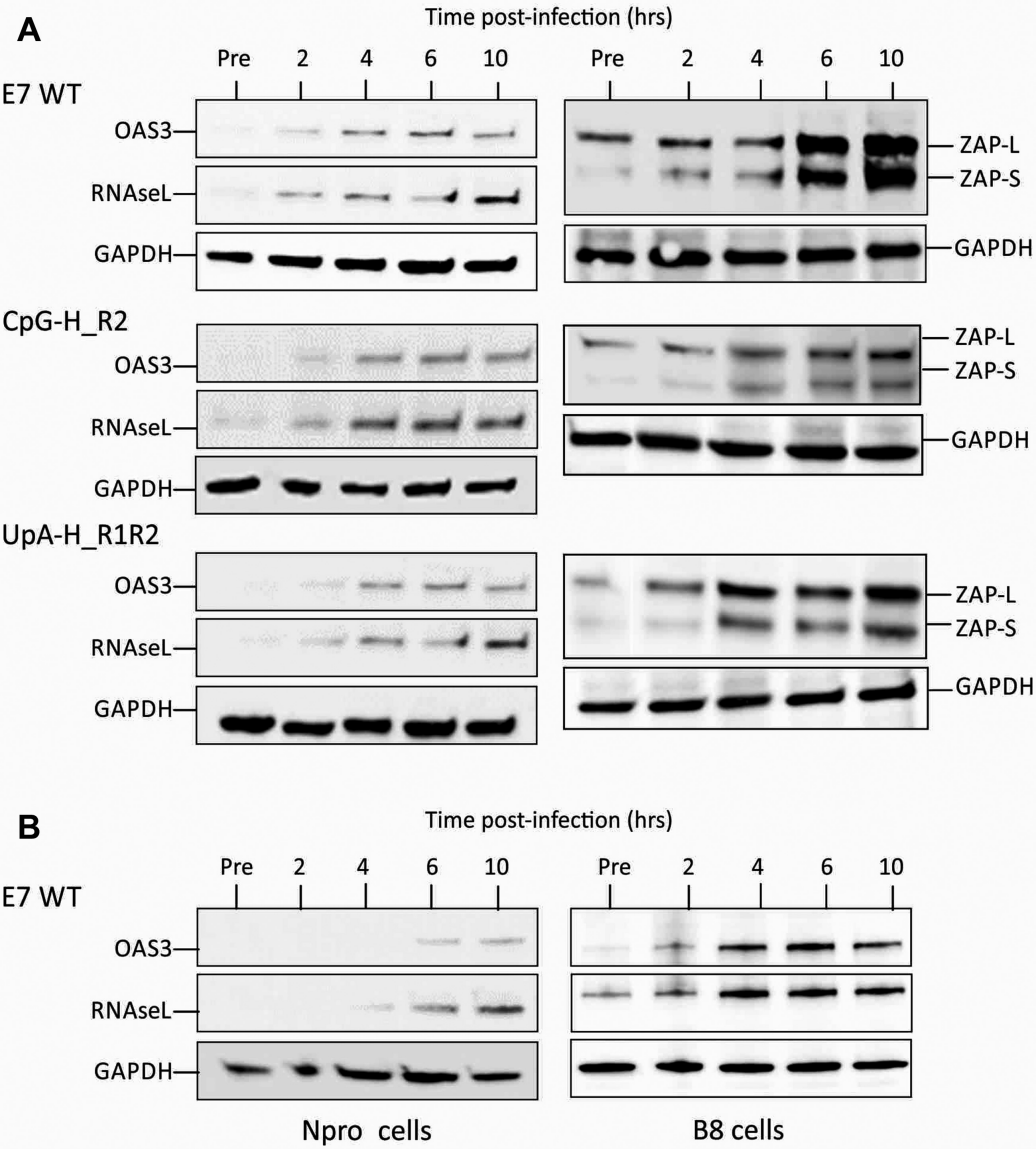
Induction of ZAP, RNAseL and OAS3 on E7 infection. (**A**) Induction of ZAP (L and S isoforms), RNAseL and OAS3 on infection with E7 over 10 h post-infection with E7 and compositionally altered mutants. (**B**) Effects of IRF3 pathway inhibition by NPro and of ZAP k/o on RNAseL and OAS3 expression in cells infected with E7 WT.

To investigate the time course for induction of these proteins on infection with E7 and whether this was influenced by virus dinucleotide composition, A549 cells were infected at an MOI of 10 with WT, CpG-H R2 and UpA-H R1R2 mutants of E7 and analysed for OAS3, RNAseL and ZAP expression every 2 h post-infection by WB (Figure 13). Over this period, expression of all three proteins substantially increased, being readily detectable at the 2-h time point. Their induction was comparable between E7 WT and the CpG- and UpA-high mutants of E7. Induction was substantially delayed and reached lower levels in cells expressing the paramyxovirus Npro protein, consistent with an IRF3-dependent activation pathway for upregulation of OAS3, RNAseL and ZAP (39). Intriguingly, constitutive expression of RNAseL was substantially greater in the B8 ZAP k/o cells pre-infection (similarly observed in B6 k/o cells; data not shown), implying some degree of regulatory interaction between what have regarded as separate antiviral pathways (discussed below).

We have previously reported that the degree of attenuation of CpG-H and UpA-H mutants of E7 differed between cultured cell types, being most marked in RD cells, and in the replicon system, less apparent in BHK and other kidney cell lines (10). Supporting an association between ZAP expression and attenuation was the observation of undetectable expression in non-activated BHK cells, in which E7 replicon attenuation was minimal (10). To investigate more systematically whether differences in constitutive or IFN-induced ZAP expression determined replicon attenuation, ZAP expression was quantified in a wide range of human, mouse and hamster of cell lines and compared to the previously determined degree of attenuation of replicons with compositionally modified 3′UTR extensions (Figures 2 and 3 and Figure supplements in (10)); Figure 12A). Cell lines with greater ZAP-L and ZAP-S expression showed a trend towards greater attenuation levels although the difference did not reach statistical significance (Figure 12C).

**Figure 14.**
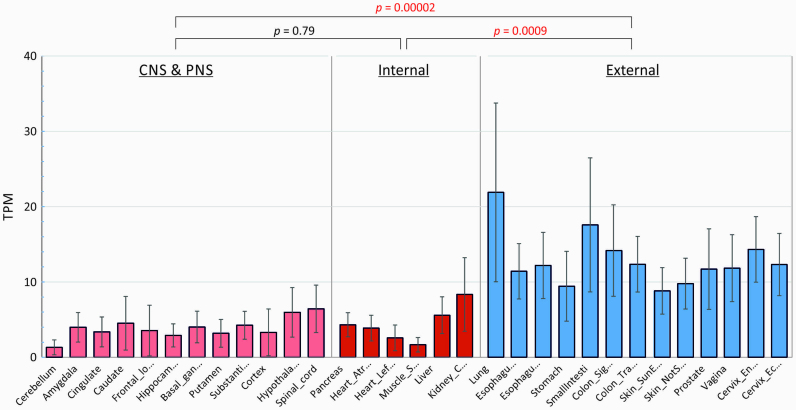
Tissue-specific expression of ZAP in different human tissues. Expression of ZAP (ZC3HAV1) mRNA sequences in different tissues quantified by RNASEQ and expressed as transcript copies/million RNAs (TPM; *y*-axis). Tissues were grouped into those most likely to encounter virus (external—lung, GI and GU tracts) and those of internal organs without direct environmental exposure (CNS/PNS and internal). Bar heights represent mean expression levels in each tissue for a mean number of 219 subjects (range 5–777); error bars show standard deviations of the mean. The significance of differences in mean values of tissues within each group was evaluated by the Kruskall–Wallace non-parametric test (*P* values shown above graph; significant values show in red).

The association of ZAP expression with CpG-induced restriction of virus replication may lead to varying degrees of attenuation *in vivo*. To investigate the extent to which constitutive ZAP and RNAseL expression varied between different cell types, we accessed the Genotype Tissue Expression (GTEx) database (https://gtexportal.org/home/) that provides quantitative data on mRNA expression levels of each cellular in over 50 different tissues and cell types drawn from large numbers of individuals (≈200) within the database file GTEx_Analysis_2016-01-15_v7_RNASeQCv1.1.8_gene_tpm. In analysing these data, we divided tissues into those most likely to be exposed to viruses (lung, gastrointestinal tract, genitourinary tract) and those of internal organs and the CNS. There was substantial constitutive expression of ZAP (Figure 14) and also of RNAseL and OAS3 (Supplementary Figure S6) in the lung and other external tissues, in all three cases significantly greater that other tissues, where expression was often at close to background levels.

## DISCUSSION

### ZAP-induced attenuation of high CpG- and UpA-viruses and replicons

The reversal of the attenuation of CpG-high mutants of E7 viruses and replicons in ZAP k/o cells is consistent with its proposed role in the recognition and control of mutants of HIV-1 with elevated frequencies of CpG dinucleotides (21). The involvement of ZAP in controlling the replication of HIV-1 adds to knowledge accumulated over the last 15 years of ZAP-mediated inhibition of alphaviruses (34,40), influenza viruses (41,42), a filovirus (43), a range of retrotranscribing viruses and retrotransposing elements (13,44–49), flaviviruses (50), even murine gammaherpesvirus 8 (51). Most of this work was performed before the RNA sequence motifs recognized by ZAP that restricted replication were identified. However, retrospectively, the preponderance of alphaviruses among the list of viruses documented to be controlled by ZAP likely reflects the greater CpG frequencies in their genomes compared to other RNA viruses, typically in the range of 0.8–0.9 observed/expected frequencies based on their G+C content. Similarly, the inhibition of Japanese encephalitis virus but not of other arthropod flaviviruses (yellow fever, dengue and Zika viruses) was proposed by the authors to be related to their differences in CpG representation (0.58 compared to 0.45 or lower for non-inhibited viruses; (50)). However, some RNA viruses appear specifically resistant to effects of ZAP, including YFV, vesicular stomatitis virus and poliovirus, even in cells overexpressing ZAP (34). An association between CpG frequencies in a range of RNA viruses and their degree of ZAP-induced attenuation has recently been experimentally demonstrated (21). The proposed role of ZAP in CpG recognition and consequent control of replication serves to unite these disparate observations collected over many years.

In that sense, the model virus E7 used in the current study typifies observations made for other RNA viruses and retroviruses. Replication of the wild-type E7 virus and replicon with their CpG O/E ratios of 0.73 (R1) and 0.32 (R2) were comparable in WT and ZAP k/o cells (Figures 1–3). Contrastingly, artificial elevation even in one relatively short region (representing <15% of the genome) served to dramatically reduce their replication, by up to 3 logs in a multistep replication assay format (Figure 1) and around 10-fold in comparably modified replicons (Figure 3). The E7 model additionally demonstrated a broader target specificity of ZAP for sequences enriched for UpA dinucleotides that were undocumented in previous studies (21,34). While elevation of UpA frequencies in E7 led to a lower proportionate degree of attenuation than CpG, it closely mirrored the reversal of attenuation observed in CpG-high mutants of E7 in ZAP k/o cells.

The possibility that pathways mediating CpG-mediated virus restriction might be shared with those restricting UpA-high viruses is supported by their extensively shared phenotypes and inferred pathway interactions reported in our current and previous studies. These include the reported independence of both CpG- and UpA-high mutants of E7 from the interferon and PKR-mediated control of virus replication (11) and caspase (10), their phenotypic reversion on treatment with the kinase inhibitor, C16 (10,11), post-entry restrictions on replication complex development and lack of co-localization with stress granules (10), and similarities in cytokine induction *in vivo* and *in vitro* (11). It is also supported by observation for 1000-fold greater binding of viral RNA with UpA-high R2 sequences compared to the WT control (Figure 4). UpA binding was indeed substantially greater than that measured to the corresponding CpG-high R2 virus mutant, with retention of 9.9 × 10^6^ RNA copies compared to 7.4 × 10^5^, despite the much greater attenuation of the latter in cell culture. Evidence for broader dinucleotide recognition by ZAP is suggested by previously reported observation of equivalent or greater attenuation of the replication of UpA-high segment 5 mutants of influenza A virus compared to CpG-high mutants in cell culture and in experimentally infected mice (12).

The mechanism by which ZAP binds to RNA and the nature of its specific targeting of CpG and UpA dinucleotides has not to date been explored at a structural level. However, the previously published structure for rat ZAP identified two potential RNA-binding cavities within a large predicted RNA-binding cleft in the zinc finger domain of the protein (52). Because this study was performed before the ligands for ZAP were characterized, it was not clear whether or if CpG dinucleotides located in the RNA-binding site nor whether ZAP’s binding specificity might extent to UpA that is also a self-complementary pyrimidine–purine pair. Intriguingly, alanine mapping showed substantial differences in the effect of amino acid changes in the two cavities on Sindbis virus recognition (which is primarily enriched for CpG). Speculatively, the two binding sites identified in the ZAP structure may possess separate specificities for CpG and UpA dinucleotides.

There is limited information currently on the potential existence of a wider ZAP recognition motif than just CpG (or UpA) that might be required for stable binding to ZAP; no specific flanking sequences were identified as influencing ZAP binding to individual CpG sites in the mutated region of HIV-1 *env* (21); on the other hand, CpG-mediated attenuation of replicon replication was greater if dinucleotides were flanked with U residues suggesting the existence of a context effect on CpG motif recognition (10). The freedom of the E7 replicon we developed to incorporate non-coding sequences in its 3′UTR (10) will enable more targeted investigations of these and other potential ZAP-binding motifs in the future.

The demonstration that the phosphorylation inhibitors SB216763, C16 and Roscovitine partly reversed the attenuated phenotype of CpG (and UpA)-high mutants of E7 (Figure 11C and (10,11)) is consistent with the previously demonstrated enhancement of ZAP function in rats by glycogen synthase kinase 3β (GSK-3β)-mediated phosphorylation of serines at positions 270, 266, 262 and 25 (38). Although C16 has been considered to be a potent and specific inhibitor of PKR-mediated phosphorylation (53), we showed it to possess a cross-inhibitory effect on GSK-3β (Figure 11B) that explains its previously noted ability to reverse CpG and UpA-mediated attenuation of E7 (Figure 11) (10,11).

### Mechanism of ZAP-induced restriction of virus replication

A plethora of interactions between ZAP and both cellular and viral cytoplasmic RNA sequences have been described. An essential co-factor for ZAP antiviral function is TRIM25, a member of the tripartite motif family of E3 ubiquitin ligases (21,54–56). While TRIM25-mediated ubiquination has been previously shown to enhance the downstream signalling of RIG-I (57,58), its interaction with ZAP is not ubiquination dependent (54,56); its ability to bind RNA perhaps serves more to allow it to co-localize with ZAP as an RNA co-receptor and provide a multimerized scaffold (55) for what remains largely uncharacterized ZAP-mediated effects on RNA degradation and virus attenuation.

Several potential mechanisms for the inhibition of virus replication by ZAP have been proposed. ZAP has been shown to repress cap-dependent translation initiation of an alphavirus (34) and HIV-1 (35), in the latter case through sequestration of the cellular translation initiation factor eIF4A. ZAP may also to target RNAs for 3′–5′ degradation to recruitment of the exosomal complex (37,48,50), and to de-cap RNAs leading to 5′-3′ degradation by the cellular Xrn1 nuclease (37,48), although whether the latter influences antiviral activity of ZAP is unclear (50). In addition to its interactions with EXOSC8, Xrn1 and other components of cellular RNA degradation pathways, ZAP, along with TRIM25, is furthermore closely associated with stress granule and P body components such as G3BP1, FXR1 and 2, ELAV2 and DDX6 (45,46), implying a central role of ZAP and other PARPs in the metabolism and turnover cellular RNAs and stress responses.

In the current study, we have used a variety of methods to investigate whether the attenuation of CpG-high and UpA-high mutants of E7 was mediated through these previously described translational inhibition or RNA degradation pathways. To investigate effects of ZAP on translation inhibition, we transfected cells with replicons with CpG and UpA-elevated 3′UTR sequences to attract ZAP. While insertion of CpG (and to a lesser extent UpA) dinucleotides had been previously shown to attenuate replication (10), neither CpG- and UpA-high mutants showed any reduction in luciferase expression post–transfection (Figure 8B). A parallel experiment was performed by modifying the composition of the luciferase gene from it is initial high CpG content (O/E ratio 1.1) in mutants with all CpG and/or UpA dinucleotides removed or minimized. In this case, the modified sequence did show higher rates of translation but this was also observed in an *in vitro* cell-free assay in which any effects of ZAP or other antiviral regulatory pathways would be unrepresented. Furthermore, an even greater enhancement of translation was observed from the codon-optimized luciferase gene even though this modified sequence possessed more CpG dinucleotides (*n* = 106) than the WT sequence (*n* = 104) (Supplementary Table S2). Further evidence for the independence of ZAP from this translational effect was provided by observations of the same differential expression of luciferase between mutants in ZAP k/o cells (Figure 9). We have previously described how the replication of HCV replicons could be enhanced by a similar removal of CpG and UpA dinucleotides from the luciferase gene (36). Replicons with CpG/UpA-low luciferase genes demonstrably showed enhanced replication kinetics potentially mediated through avoidance of ZAP. However, the CpG/UpA-low mutant replicon also showed enhanced translation where replication was prevented, either in a non-permissive cell line or in the presence of GnHCl (Figure 9B). Similar expression differences were observed in ZAP k/o cells. E7 possesses a type 1 internal ribosomal entry sites that is dependent on eIF4A for translation initiation; HCV possesses a type III that is eiF4A-independent (59)—neither appeared to be influenced by ZAP-mediated targeting of the template RNA in the experimental system used in the current study.

We further investigated whether ZAP targeting of high CpG and UpA RNA sequences led to greater degradation through previously documented exosomal or other cleavage pathways (37,48–50). However, transfected CpG- or UpA-modified E7 replicons showed the same decay rates as WT sequences post transfection in the absence of replication (Figure 10C). Similarly, arrest of actively replicating virus by addition of GnHCl led only the slow decay of WT and compositionally modified E7 RNA levels over the following 24 h (Figure 10B). Finally, we found no evidence for specific targeting and degradation of regions of CpG or UpA modified sequence in infected cells (Figure 10A). These largely negative findings contrast with previously published data showing reduced expression levels of CpG-modified HIV-1 mRNAs compared to WT transcripts (21). However, this apparent difference from E7 may originate from the different RNA configurations of HIV-1 and enteroviruses and the activity of virally encoded degradation pathway antagonists. XRN1 5′-3′ degradation depends on de-capping to expose a naked 5′end while recruitment of the exosomal complex and subsequent degradation depends on initial removal of the poly(A)-tract by poly(A) ribonuclease (PARN) to the RNA 3′ end. Both of these RNA modifications are found in mRNAs (and viral RNAs) of HIV-1, other retroviruses and indeed almost all DNA viruses. However, the 5′end of E7 is covalently coupled to the virally encoded VpG rather than capped and likely prevents XRN1-mediated 5′-3′ cleavage. Although not firmly attributed to ZAP at that time, the previously reported inhibition of (cap-dependent) translation of Sindbis virus and lack of effect on VpG-terminated and IRES-dependent translation of the encephalomyocarditis virus in dendritic cells (60) is consistent with the hypothesis. Concerning the possibility of 3′→5′ degradation, enteroviruses possess a poly(A) tail and may potentially be targeted by the cellular exosomal complex. However, infections with poliovirus (and potentially other enteroviruses) accelerate the degradation of the 3′deadenylase complex component Pan3 (61) and prevent the de-adenylation required for 3′-5′ exosomal degradation. E7 may be similarly resistant.

Interactions between ZAP and proteins associated with stress granule (SG) formation has led to the proposal that ZAP may exert its antiviral or retrotransposon activities through sequestration of viral RNA in SGs (45). While ZAP was reported to co-localize with the LINE-1 element in SGs, our previous study demonstrated no evidence for co-localization of E7 RNA (10), perhaps a consequence of its antagonism of exosomal recruitment and XRN1-mediated RNA degradation that typically localizes to SGs (37,62) or degradation of SG components such as G3BPs (63 ). Analysis of ZAP expression and E7 RNA detected by FISH (Figure 5B) did not provide any strong evidence for co-localization that might have been expected if ZAP directly interacted with the E7 replication complex. An intriguing additional mechanism for the antiviral function of ZAP might be through pathway signalling. We observed marked re-localization of ZAP to the nucleus on infection with E7, consistent with the previous detection of two nuclear localization sequences and a nuclear export signal in ZAP and a CRM1-dependent shuttling mechanism (64). Its localization to nucleolar areas associated with DNA transcription is consistent with a role for ZAP in as yet unidentified pathway activation in infected cells that may amplify or complement existing IFN-induced antiviral responses.

The use of virus models, such as influenza A virus, in which individual viral genes are expressed as separate mRNAs, and a better understanding of how ZAP-activated pathways may be antagonized by the virus will be required to more directly address mechanistically how CpG and UpA-modified RNA sequences are targeted by ZAP and potentially other recognition systems such as OAS/RNAseL.

### RNAseL-mediated changes in virus attenuation

The second principal finding in the current study was the unexpected but consistently observed separate reversal of CpG- and UpA-mediated virus attenuation in RNAseL and OAS3 knockout cells. Reversal of attenuation was observed in independently generated CRISPR k/o cell lines (E10 and F7) and in both replicating virus and replicons (Figures 1 and 3). Furthermore, separate knockout of OAS3, but not OAS1, reproduced the attenuation pattern of E10 and F7 cell lines, substantiating the RNAseL association and suggesting that the recognition of CpG- and UpA-enriched sequences lay in upstream OAS-mediated recognition of RNA. The potential role of OAS3 in the recognition of CpG- and UpA-high mutants is consistent with its demonstrated preferential role in mediating RNAseL responses to a wide range of virus infections, and the frequent inactivity of OAS1 (and OAS2) in antiviral defence (25–27). The pronounced antiviral effect of OAS3 on Sindbis virus (25) that possesses a relatively high CpG content (O/E ratio 0.90) indirectly supports the proposed sequence specificity of this antiviral pathway.

However, whether and how CpG- or UpA-high sequences might be first recognized by OAS3 and presumably degraded by RNAseL is far from clear. The first problem is that the target of OAS3 (and other OASs) is dsRNA; OAS3 furthermore appears specifically designed to target long dsRNA or poly(I:C) sequences (50 base pairs or longer) through trimerization of the RNA recognition domains, D1–D3 (65). Its specificity for dsRNA was demonstrated by the inability of chemically synthesized ssRNA to activate OAS3 (or OAS1) (65). Furthermore, the conventional view is that there is no sequence specificity of dsRNA recognition by OASs, seemingly at odds with the apparent specificity of OAS3 recognition for high CpG and UpA sequences. It has however recently been found that certain sequence motifs, such as the rather loose WpWpN^9^pWpG sequence motif, may lead to more potent binding and activation of OAS1 (66). With this precedent, perhaps OAS3 may similarly possess recognition motifs in dsRNA that are preferentially activated by high CpG- or UpA-containing sequences.

The second intriguing but conflicting observation is that the RNAseL- and OAS3-associated phenotypic reversion occurs in the same system where attenuation of CpG-high mutants can be entirely reversed in ZAP k/o cells. For example, extensive replication of high CpG- and UpA- high mutants of E7 in cells co-expressing ZAP in OAS3 k/o cells can be directly visualized by FISH (Figure 6). How can OAS3/RNAseL also reverse their phenotype unless there is some commonality with ZAP in their restriction pathways? There is little or no evidence for such interactions in the current literature. For example, while ZAP appears to interact with a vast range of other cellular proteins, with 78 co-precipitating with the short isoform of ZAP (45), that list did not include any isoform of OAS3 or RNAseL. However, preliminary evidence for the existence of a cross-regulatory interaction between ZAP and RNAseL/OAS3 might include the observation that ZAP k/o leads to much greater constitutive expression of RNAseL (Figure 13B). Conversely, OAS3 k/o leads to an upregulation of ISG expression activated through MAVS (67). However, further work is needed to disentangle direct effects between these antiviral pathways and effects mediated through differences in interferon induction and secondary activation of ZAP, OASs and RNAseL expression.

A further curious observation was the observation of complete reversion of CpG- and UpA-high E7 mutants in OAS3 k/o cells, but only a partial reversion of the replication of these mutants in RNAseL k/o cells. This cannot be the result of RNAseL only being partly knocked out—the E10 cell line showed no detectable expression of RNAseL constitutively or after IFN stimulation by western blot (Figure 12B) and there was ablation of all three alleles of the RNAseL gene in the (triploid) A549 cell line. The observation leaves open the possibility that the 2–5A may exert its effects on other elements of the innate immune system (for example, 2–5A is documented to downregulate type I topoisomerase; (68)). Alternatively, OAS3 may itself possess antiviral functions analogous to those of the OAS-like paralogue, OASL which is unable to produce 2–5A. Both the long isoform of OASL and OAS3 have nuclear localization signals; OASL may influence cellular transcription through interaction with methyl CpG- binding protein 1 (69). These observation suggest potential roles for OAS3 in cellular immunity unrelated to RNAseL activation.

### Evolutionary perspectives on CpG and UpA dinucleotide suppression in RNA viruses

The demonstrated antiviral effect of ZAP on alphaviruses (34,40), other RNA viruses (41–43,50) and retro-transcribing viruses and transposable elements (13,44–49), has been consistently interpreted as evidence for the existence of a separate, IFN-independent mechanism to control the replication of virus infections in mammalian cells. Clearly, ZAP-mediated restriction of virus replication, however it might work, is highly effective in viruses with artificially elevated frequencies of CpG (and UpA) frequencies (10–12,21) but the pathway typically has no effect on the replication of native, unmodified versions of these viruses (for example E7 in Figures 1 and 3; (21,34)). Given the flexibility of the genetic code and the ease in which CpG (and UpA) frequencies can be modified without affecting protein coding, ZAP might seem a rather useless form of antiviral defence. Indeed, the pervasive suppression of CpG frequencies in most vertebrate RNA viruses and retroviruses (7–9) to levels that match those of cellular mRNAs might be interpreted as a simple adaptive change to avoid recognition by ZAP and potentially other cellular pathways of compositionally abnormal RNA sequences. The processes by which viruses adapt to and co-exist with their hosts over long periods clearly provide them with the opportunity to finesse numerous aspects of the mononucleotide and dinucleotide composition to maximize fitness (70)

ZAP is a member of a larger family of poly-adenosine diphosphate ribosyl transferase proteins (PARPs) that play roles in DNA and RNA metabolism and repair (reviewed in (71)). Some possess zinc finger domains that bind nucleic acid sequences, which in the case of ZAP, likely mediates its ability to detect high concentrations of CpG (and UpA) dinucleotides in RNA sequences and to restrict virus replication, however that might be achieved. While the functions of PARPs are not primarily antiviral, several others including PARP4, PARP9, PARP14 and PARP15 in addition to ZAP show evidence for positive selection and rapid adaptive sequence change (72,73) that typify cellular proteins involved in cellular (innate) antiviral defence. Their accelerated evolution reflects the arms race between the host and the virus, with its vast and rapidly innovating battery of viral antagonistic pathways (1). While it is intriguing to imagine what the target specificities of these additional potential antiviral PARPs might be, none possess zinc finger domains that are typically associated with nucleic acid binding.

The complexity of the interactions between ZAP and RNA viruses is exemplified by alphaviruses. Unlike most other RNA viruses, currently described species do not substantially suppress CpG frequencies; consequently their replication appears to be restricted by ZAP (34,40). Variability of CpG composition and ZAP susceptibility is also apparent in the arthropod-borne flaviviruses (50). Why have these viruses not simply suppressed CpG frequencies to avoid recognition and restriction by ZAP? Is their optimization of genome composition more targeted towards insect-specific fitness optimization that outweighs their consequent impaired replication in mammalian and avian hosts in which ZAP-mediated restriction is active? Alternatively and more intriguingly, is CpG representation in viruses optimized for aspects of evolutionary fitness that do not precisely coincide with maximized replication rate? For example, accelerated replication of Sindbis virus and other alphaviruses in human and other mammalian hosts might reduce their overall fitness if their increased replication and pathogenicity truncated the duration of transmissibility (e.g. by increasing their case fatality rate). Perhaps the various degrees of CpG suppression manifested by vertebrate RNA viruses represent different balances of infectiousness and persistence to maximize evolutionary (transmission) fitness. ZAP may indeed have been the unwitting partner in a pathway exploited by viruses to finely tune their replication to maximize their evolutionary fitness. The extraordinary effects of artificial manipulation of CpG and UpA frequencies in model viruses such as E7, HIV-1 and IAV may have helped to identify the existence of such cellular pathways but perhaps not their true role in viral pathogenesis.

### Development of attenuated vaccines by modification of CpG and UpA dinucleotide frequencies

Several groups have used mutagenesis strategies, such codon de-optimization (15) and the use of disfavoured codon pairs (14,16,74), in an attempt to impair translation efficiency and to generate safer, non-reverting attenuated virus vaccines. As extensively discussed previously (18–20,75,76) however, effects of these mutagenesis strategies are more likely mediated through the unintended increase in CpG and UpA frequencies in the modified sequences, since both dinucleotides are suppressed in typical mammalian codon repertoires and at codon junctions (19). Potentially, therefore, vaccine attenuation depends on the active suppression of replication by ZAP and separately by OAS3/RNAseL rather than the viruses being intrinsically defective. In the current study, we demonstrated an association between constitutive expression of ZAP-L and ZAP-S with the degree of attenuation of CpG-enriched replicons (Figure 12C). Most strikingly, the least degree of attenuation was observed in BHK cells, which express no detectable ZAP in the absence of interferon activation (Figure 12A). Putting this together, the efficacy of attenuation might depend critically on the degree of cellular ZAP and OAS3/RNAseL expression in the infected tissue. As demonstrated, this is far from uniform, with particularly low expression of both ZAP, RNAseL and OAS3 throughout the CNS (Figure 14; Supplementary Figure S6), consistent with the previously reported differential expression of ZAP in mouse lymphoid and CNS tissue (77 ). A theoretical concern but nevertheless supported by the analysis presented in the current study is that engineered vaccines for neurotropic viruses, such as poliovirus, Japanese encephalitis virus and enterovirus A71, may well show restricted replication at sites of entry but may lose that attenuation once they enter the CNS. Conceivably, for example, an attenuated poliovirus vaccine may show a similar propensity to induce paralytic disease as the WT virulent strains.

The other danger arises from the possibility that efficacy of ZAP- and OAS3/RNAseL-mediated attenuation may vary between individuals. The *hOAS3* gene locus is quintessentially polymorphic in human populations with several SNPs associated with differences in susceptibility to tick-borne encephalitis and dengue viruses, enterovirus A71 and outcome of hepatitis B virus infections (78–82). As functioning ZAP and OAS3/RNAseL pathways are both required for high CpG-/UpA-mediated virus attenuation, variability in OAS3 function may severely impact upon the protective efficacy of such engineered vaccines. Reassurance on efficacy and safety might include focussed investigations of potential tissue-specific and population-based differences in vaccine attenuation before they are widely used in human and veterinary practice.

## Supplementary Material

gkz581_Supplemental_Files

## Notes

Present address: Isabelle Dietrich, The Pirbright Institute, Ash Road, Pirbright, Woking, GU24 0NF, UK.

Present address: Kinda Alshaikhahmed, Faculty of Infectious and Tropical Diseases, London School of Hygiene and Tropical Medicine, Keppel Street, London WC1E 7HT, UK
