# Supplementary material for: The role of ZAP and OAS3/RNAseL pathways in the attenuation of an RNA virus with elevated frequencies of CpG and UpA dinucleotides
Source: Nucleic Acids Res. 2019 Jul 5;47(15):8061–83. doi: 10.1093/nar/gkz581 (PMC6735852; doi:10.1093/nar/gkz581)
Supplement: gkz581_Supplemental_Files [file gkz581_supplemental_files.zip › Supplementary Data_revised_v2.pdf]

TABLE S1

SEQUENCES OF PRIMERS USED IN THE STUDY

gRNA for CRISPR cell lines (5'-3')

ZAP	5'-CGACGTGGTCCAGAACATCC-3'
RNAseL	5'-TTAATTTCCAGGAAGAGGAA-3'
OAS1	5'-TCATCCGCCTAGTCAAGCAC-3'
OAS3	5'-AATTGCTGACCATCTTCGCC-3'

Primers for sequencing modified cell lines

ZAP F	5'-GTCGGCAGCAGATTTGTAACC-3'
ZAP R	5'-ACGGCAAGGTAGAGTGATTATGG-3'
RNAseL	F 5'-CTTCGTTGCCAGGTGGAATGT-3'
RNAseL	R 5'-ATCCTCCTTTGTCTTTCGCCTC-3'
OAS3 F	5'-TGTTCTTGGAACAGGCTTGGA-3'
OAS3 R	5'-GTACATGTAGCATTTTGCTTTGGC-3'
OAS1 F	5'-TTGTCCCCTCAGAGTGACTGAA-3'
OAS1 R	5'-GGCATGCTACTCGTTGCAGAT-3'

E7 RNA quantitation:

EQ1:	5'-ACATGGTGTGAAGAGTCTATTGAG-CT-3'
EQ2:	5'-CCAAAGTAGTCGGTTCCGC-3'
Probe:	[6FAM]TCCGGCCCCTGAATGCGGCTAAT[TAM]

ZAP mRNA detection

Mamm_ZAP_s 574 CTGATGGACAGRAAGGTGYTGGC

Mamm_ZAP_as 759 GAABCGRTCTCTRCTYTTGCTTCT

Primers for stability of the viruses:

R2_WT_s	5598	TGGAGCTGACACTACTCAAGTTAAACC
R2_CDLR_s	5598	5'-TGGAACTCACACTCCTCAAGTTAAACC
R2_CpH-High_s	5598	5'-TCGAGCTGACGCTACTCAAGTTAAACC-3'

R2_UpA-High_s	5598	5’-TAGAGCTTACACTACTTAAGTTAAACC-3’
R2_WT_a	6022	5’-ACTTGGCTCCAGCTTGGTTTTACTGG-3’
R2_CDLR_a	6022	5’-ACTTGGCTCAAGCTTGGTCTTACTAG-3’
R2_CpH-High_a	6022	5’-GCTCGGCTCGAGCTTCGTTTTACTCG-3’
R2_UpA-High_a	6022	5’-ACTAGGCTCTAGCTTAGTTTTACTAG-3’

TABLE S2

Dinucleotide composition and coding metrics of firefly luciferase gene and mutants

		Totals		O/E ratios		Coding metrics[1]		
**Sequence**	**G+C**	**CpG**	**UpA**	**CpG**	**UpA**	**ENc**	**CPB**	**CAI**
Firefly WT	45.2%	104	88	1.205	0.693	57.7	-0.10	0.72
UpA-Low	48.4%	104	21	1.047	0.187	52.6	-0.07	0.77
CpG-Zero	40.0%	0	88	0	0.578	44.2	0.10	0.75
CpG/UpA-Low	43.1%	0	21	0	0.153	42.9	0.11	0.80
Codon Opt.	59.6%	106	32	0.706	0.475	27.4	-0.01	0.96

[1]Coding metrics: ENc: effective number of codons; CPB: codon pair bias; CAI: codon adaptation index

TABLE S3

Bonferroni's multiple comparisons test of co-localisation of E7 RNA and ZAP between E7 ET and compositionally modified mutants.

**Comparison**	**Mean Diff.**	**95% CI of diff.**	***p* value**
WT E7 vs. CpG-H	-0.1023	-0.23-0.02	0.1487
WT E7 vs. CpG-H, R1R2	-0.1473	–0.27-0.02	**0.0198**
WT E7 vs. UpA-H	-0.1176	-0.24 to 0.01	0.0763
WT E7 vs. UpA-H,R1R2	-0.1575	-0.28 to -0.03	**0.0124**

Fig S1. Verification of knockout of CRISPR targeted genes in cell lines

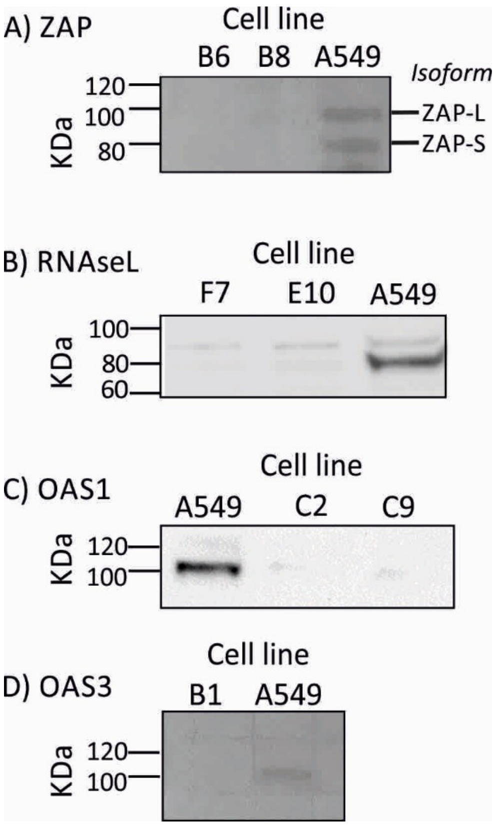

Detection of ZAP, RNAseL, OAS1 and OAS3 by immunoblot in parental A549 cells and k/o cell lines used in the current study. Target proteins were stained using RNAseL, ZCCHV (ab154680), OAS1 (ab86343) and OAS3 (ab154270)-specific antibodies.

Fig S2. Effect of ZAP, RNAseL and OAS1/2 expression on

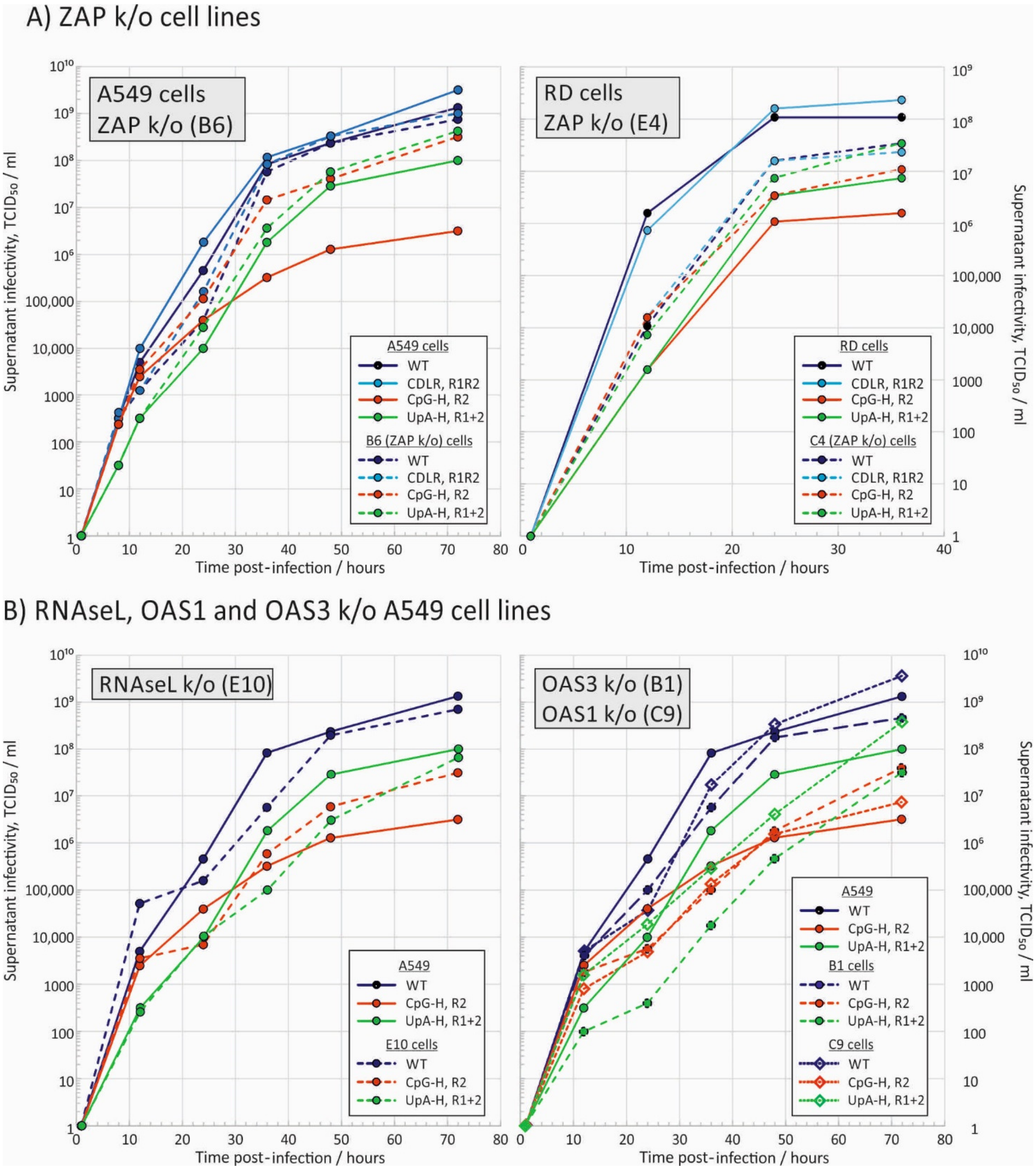

Side-by-side comparison of WT and mutant E7 replication in A549 or RD cells with that of ZAP (B6, E4), RNAseL (E10), OAS3 (B1) and OAS1 (C9) k/o cells. This format directly demonstrates effects of k/o on WT and each mutant in the same graph frame. Data points were calculated as described in the legend for Fig. 1.

Fig S3. Replication of WT and compositionally modified mutants of E7 In the B8 ZAP k/o cell line.

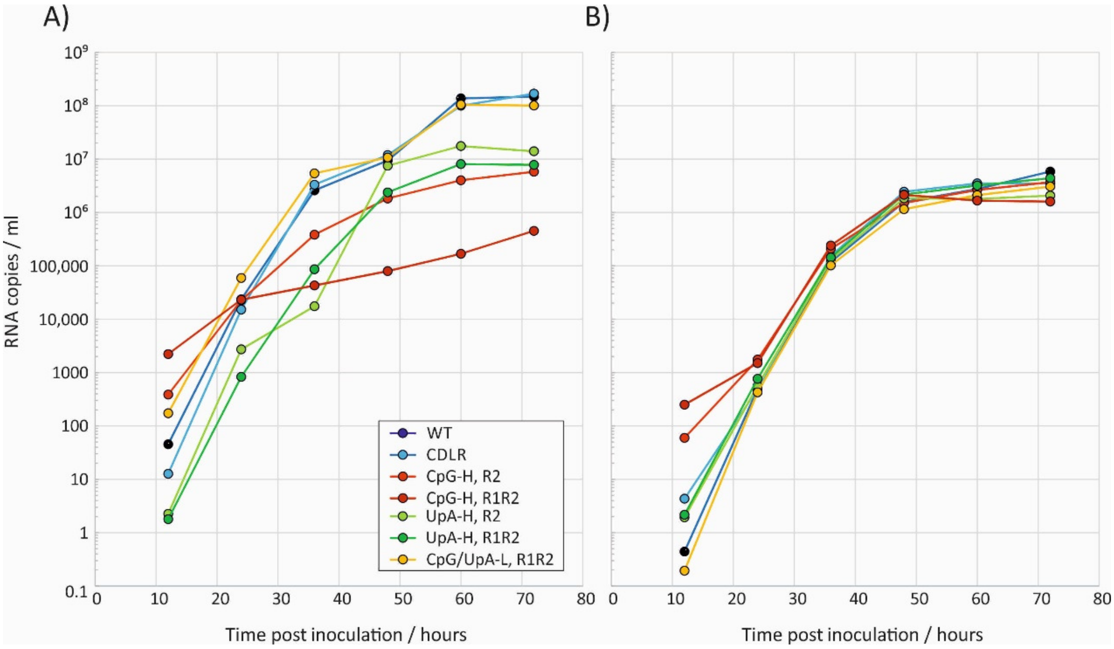

Multi-step replication kinetics of E7 WT and compositionally altered mutant viruses in (A) A549 cells and (B) the B8 ZAP k/o cell line. Cells were infected with a MOI of 0.01 and supernatant sampled and assayed for E7 RNA viral loads at different time points after infection. Graph lines show mean values of supernatants from two biological replicates. This analysis includes replication kinetics of the CpG-high R1R2 mutant that could not be assayed by infectivity measurements (as used in Fig. 1)

Fig. S4. Colocalisation of ZAP and E7 RNA in infected A549 celLs

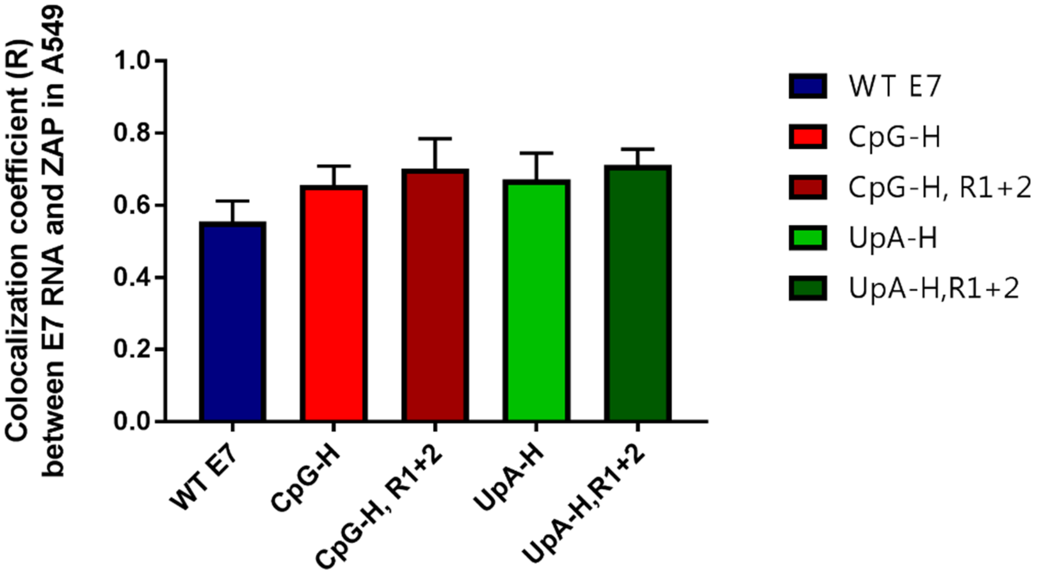

Coefficients of correlations are presented. The range is from −1, complete avoidance of two markers, 0: random localisation and +1, complete co-localisation.

Fig. S5. Luciferase expression in compositionally modified E7 replicons in a cell free assay

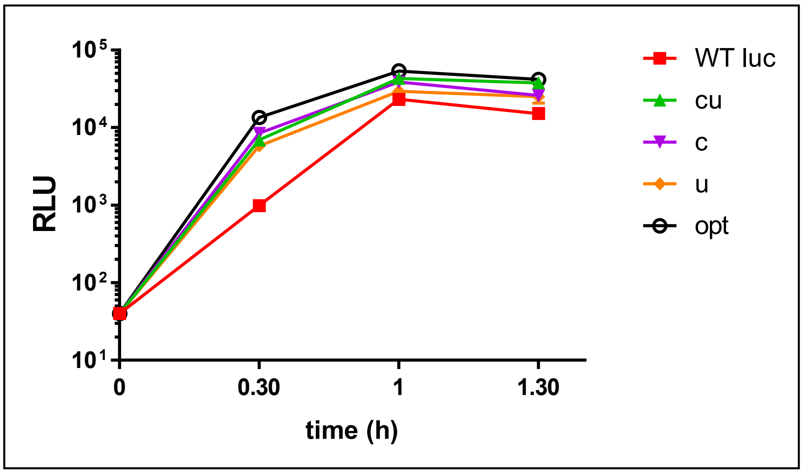

Translation of E7 replicons with compositionally modified luciferase genes in wheat germ extract; translation was recorded by luciferase expression at 30 – 90 minutes post-initiation. Key: cu: CpG/UpA-L; c: CpG-zero; u: UpA-L; opt: codon optimised.

Fig. S6. Tissue-specific expression of ZAP-L and RNAseL in different human tissues

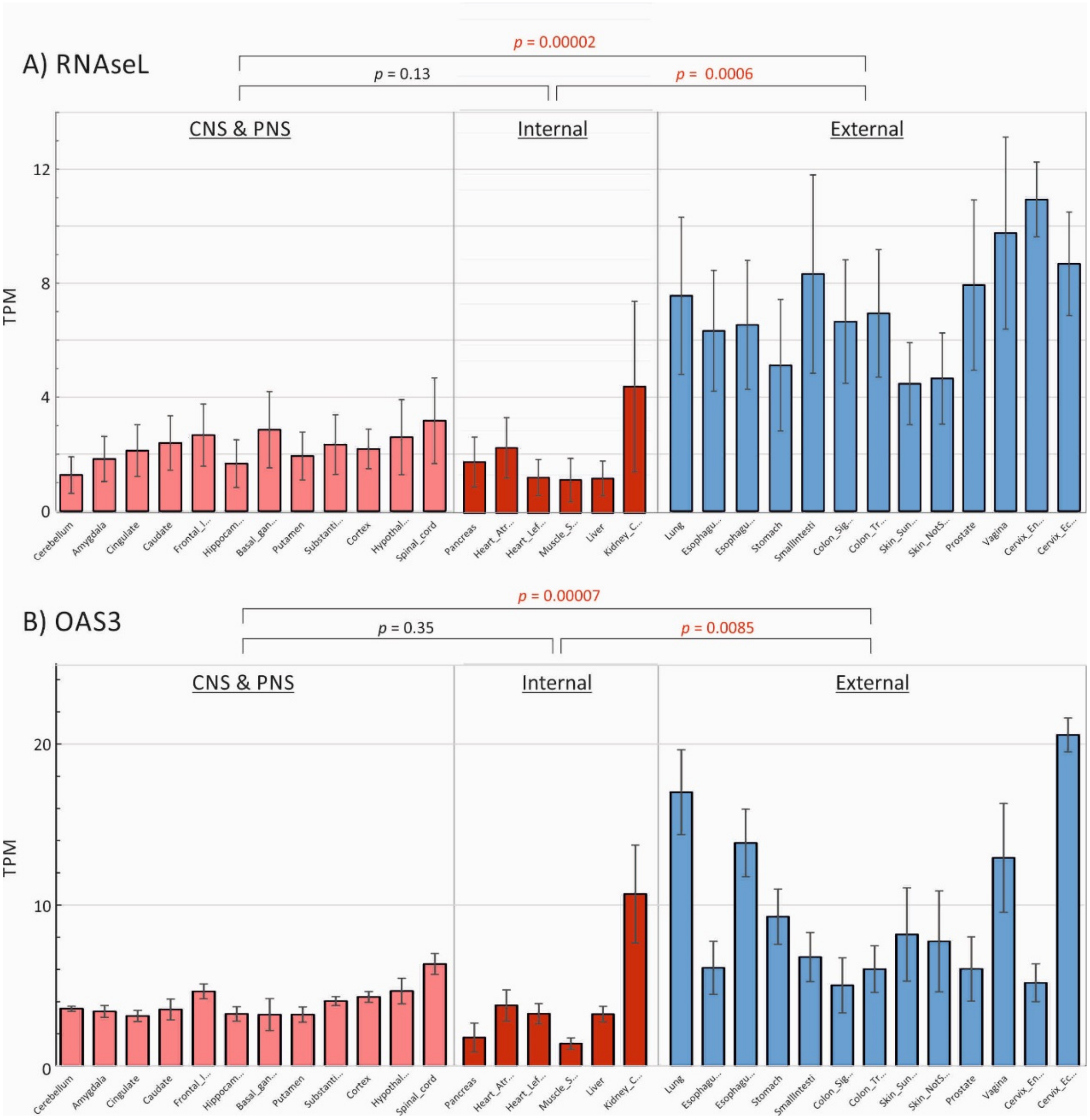

Expression of RNAseL and OAS3 mRNA sequences in different tissues quantified by RNASEQ and expressed as transcript copies / million RNAs (TPM; y-axis). Tissues were grouped into those most likely to encounter virus (external - lung, GI and GU tracts) and those of internal organs without direct environmental exposure (CNS/PNS and internal). Bar heights represent mean expression levels in each tissue for a mean number of 219 subjects (range 5-777); error bars show standard deviations of the mean. The significance of differences in mean values of tissues within each group was evaluated by the Kruskall –Wallace non-parametric test (p values shown above graph; significant values shown in red).
